# The chemokines CXCL8 and CXCL12: molecular and functional properties, role in disease and efforts towards pharmacological intervention

**DOI:** 10.1038/s41423-023-00974-6

**Published:** 2023-02-01

**Authors:** Seppe Cambier, Mieke Gouwy, Paul Proost

**Affiliations:** Laboratory of Molecular Immunology, Rega Institute, Department of Microbiology, Immunology and Transplantation, KU Leuven, Leuven, Belgium

**Keywords:** CXCL8, CXCL12, atypical chemokine receptor, GPCR, glycosaminoglycan, Chemokines, Inflammation, Neutrophils, Leukopoiesis, Cancer

## Abstract

Chemokines are an indispensable component of our immune system through the regulation of directional migration and activation of leukocytes. CXCL8 is the most potent human neutrophil-attracting chemokine and plays crucial roles in the response to infection and tissue injury. CXCL8 activity inherently depends on interaction with the human CXC chemokine receptors CXCR1 and CXCR2, the atypical chemokine receptor ACKR1, and glycosaminoglycans. Furthermore, (hetero)dimerization and tight regulation of transcription and translation, as well as post-translational modifications further fine-tune the spatial and temporal activity of CXCL8 in the context of inflammatory diseases and cancer. The CXCL8 interaction with receptors and glycosaminoglycans is therefore a promising target for therapy, as illustrated by multiple ongoing clinical trials. CXCL8-mediated neutrophil mobilization to blood is directly opposed by CXCL12, which retains leukocytes in bone marrow. CXCL12 is primarily a homeostatic chemokine that induces migration and activation of hematopoietic progenitor cells, endothelial cells, and several leukocytes through interaction with CXCR4, ACKR1, and ACKR3. Thereby, it is an essential player in the regulation of embryogenesis, hematopoiesis, and angiogenesis. However, CXCL12 can also exert inflammatory functions, as illustrated by its pivotal role in a growing list of pathologies and its synergy with CXCL8 and other chemokines to induce leukocyte chemotaxis. Here, we review the plethora of information on the CXCL8 structure, interaction with receptors and glycosaminoglycans, different levels of activity regulation, role in homeostasis and disease, and therapeutic prospects. Finally, we discuss recent research on CXCL12 biochemistry and biology and its role in pathology and pharmacology.

## Introduction

Chemokines or *chemo*tactic cyto*kines* are a family of small and mostly secreted proteins, with a molecular mass of about 7–14 kDa. They regulate directional leukocyte migration and activation during inflammatory and homeostatic processes in a time- and site-dependent manner. Moreover, chemokines play a role in angiogenesis, tumor growth and metastasis, hematopoiesis, organogenesis, cell survival, proliferation, differentiation, and many other processes [1, 2].

Chemokines are divided into four subfamilies based on the relative position of the first two of four conserved cysteine residues. Two disulfide bridges, between the first and third and the second and fourth cysteine residue, stabilize the chemokine tertiary structure, composed of a highly conserved three-stranded β-sheet/α-helix structural fold. CC chemokines possess two adjacent cysteines in the NH_2_-terminal region, whereas one or three other amino acids separate the first two cysteine residues in CXC and CX_3_C chemokines, respectively. Only one CX_3_C chemokine is known, being the membrane-bound CX_3_CL1 (fractalkine). XC chemokines, XCL1 (lymphotactin α) and XCL2 (lymphotactin β), lack the first and third cysteine residue. CXC chemokines encompass both ELR^+^ (e.g. CXCL8) and ELR^−^ (e.g. CXCL12) chemokines, based on the presence or absence of a Glu (E) – Leu (L) – Arg (R) tripeptide motif NH_2_-terminally from the first cysteine. ELR^+^ chemokines are predominantly neutrophil attractants, whereas ELR^−^ chemokines regulate monocyte, basophil, eosinophil, lymphocyte, and natural killer cell chemotaxis [3]. Biologically, chemokines are classified as inflammatory and homeostatic chemokines. Inflammatory chemokines (e.g. CXCL8) coordinate the directional chemotaxis of leukocytes to inflammatory sites (infection & tissue injury). Their production is induced in response to endogenous (e.g. pro-inflammatory cytokines) or exogenous (e.g. pathogen-associated molecular patterns) inflammatory triggers. Homeostatic chemokines (e.g. CXCL12) are constitutively expressed and regulate homeostatic basal migration of immune cells within and between lymphoid organs, blood, and peripheral tissues establishing immune surveillance. Moreover, they regulate hematopoiesis in the bone marrow and thymus and play a role in several developmental processes. Some chemokines are characterized with both homeostatic as inflammatory functions [1, 3].

Chemokines interact with two essential partners to exert their chemotactic activity in vivo: endothelial and tissue glycosaminoglycans (GAGs) and seven-transmembrane spanning chemokine receptors. GAGs are large, linear, and negatively charged polysaccharides and usually part of larger proteoglycan structures present on cell surfaces, in the extracellular matrix, or glycocalyx [4]. The presentation of positively charged basic chemokines on negatively charged GAGs is crucial for immobilization of chemokines on the endothelial surface [5, 6]. This prevents diffusion by the blood stream so that a chemokine concentration gradient toward the inflammatory stimulus is established. In addition, it enables binding of the chemokines to their chemokine receptors expressed on different leukocytes, resulting in leukocyte extravasation. Interstitial chemokine gradients established by GAG binding further guide leukocyte migration to, and activation within, the inflamed tissue. Chemokine G protein-coupled receptors are categorized according to the structure of the chemokine ligands (CCR, CXCR, CX_3_CR and XCR) [3]. Moreover, atypical chemokine receptors (ACKRs) interact with chemokines. ACKRs cannot signal via G proteins and therefore mainly act as “silent” scavenging receptors (interceptors) dampening immune responses by binding, internalizing, and degrading chemokines, but can also promote chemokine transcytosis and exert important signaling functions [3, 7]. The categorization nomenclature and an overview of the almost 50 human chemokines, their (atypical) receptors, and target cells is reviewed elsewhere [3, 7, 8].

In this review, we aim to provide a comprehensive overview of the most potent human neutrophil-attracting and -activating inflammatory chemokine interleukin-8 (IL-8) or C-X-C motif chemokine ligand 8 (CXCL8). Its discovery and production, structural interactions with GAGs and receptors, functions, activity regulation, role in disease as well as therapeutic perspectives will be discussed. Recent advances have been summarized in Box 1. Additionally, CXCL8 has an interesting connection with the traditionally considered homeostatic chemokine stromal cell-derived factor 1 (SDF-1) or CXCL12. While CXCL12 is retaining neutrophils in the bone marrow, CXCL8 induces neutrophil mobilization to blood. Conversely, CXCL12 is known to synergize with CXCL8 and granulocyte chemotactic protein (GCP)-2 (the likely functional murine homolog of human CXCL8) in the chemotaxis of neutrophils in vitro and in vivo toward tissues, respectively. Hence, CXCL12 can also play an inflammatory role. In the final part of this review, we will provide an update on the research progress that was made in the field of CXCL12 since the review manuscripts published in 2018 describing the structural and functional features as well as the pathological roles of CXCL12 [9, 10].

Box 1 Novel research in the field of CXCL8

**Table:** 

		Ref.
Structure & expression	First cryo-electron microscopy structures of dimeric & monomeric CXCL8-activated human CXCR2	[35]
	Murine bone marrow macrophages & human monocytes do not express ACKR1	[39]
Function	ACKR1 creates a chemokine “depot” by presenting CXCL2 in endothelial cell junctions which promoted self-guided neutrophil transendothelial migration in mice	[47]
	ACKR1 retention of CXCL1 in endothelial cell junctions induced CXCR2 desensitization & reversed transendothelial migration of neutrophils in aged mice, which resulted in remote organ damage	[51]
	GAGs may provide an immobilized CXCL8 reservoir, in equilibrium with free CXCL8 in solution that interacts with its receptors (chemokine cloud model)	[6, 67]
Activity regulation	CXCL8(9–77) may be the most potent neutrophil-attracting CXCL8 proteoform in vivo	[93]
Disease & therapy	Neutrophil diversity and plasticity underlie the tumor-promoting or tumor-suppressing effects of neutrophils in the tumor microenvironment	[107]
	Tumor-produced CXCR1/CXCR2 ligands induce the release of neutrophil extracellular traps, which can wrap and coat tumor cells shielding them from immune cytotoxicity by CD8^+^ T-lymphocytes and natural killer cells	[110]
	CXCL8 is associated with resistance to anti-tumor (immuno)therapy. Combination therapy with CXCL8-CXCR1/CXCR2 inhibitors may provide further benefit in cancer treatment	[112, 113]
	CXCL8 production by endothelial colony-forming cells might contribute to neutrophil infiltration in idiopathic pulmonary fibrosis	[133]
	Neutrophils in inflamed joints of juvenile idiopathic arthritis (JIA) patients display a hyperactivated phenotype. A complex intertwining between innate and adaptive immunity probably drives JIA	[142]
	CXCL8-CXCR1/CXCR2 may induce podocyte damage in type 2 diabetic kidney disease	[160]
	Mucosal CD14^+^ monocyte-like cells induced CXCL8 in colonic memory CD4^+^ T-lymphocytes, showing crosstalk between innate and adaptive immunity in ulcerative colitis	[165]
	Key role for CXCL8 & neutrophils in the pathogenesis of severe COVID-19, with improvements in clinical outcomes of patients treated with a CXCR1/CXCR2 antagonist	[166, 168, 169, 187]
	Anti-CXCL8 autoantibodies might reduce the severe systemic inflammation associated with neutrophil activation in COVID-19	[170]
	Blocking CXCR1/CXCR2 signaling reduced NET formation, tissue injury & mortality without impairing bacterial clearance in septic mice	[171]

## The inflammatory chemokine CXCL8

### Discovery & cellular sources of CXCL8

In 1987–1988, different independent research groups identified natural IL-8 (later named CXCL8 upon establishment of the systematic nomenclature for chemokines [11]) from stimulated cell culture supernatants as a low molecular mass protein with potent neutrophil chemoattractant properties [12]. CXCL8 was purified and described as a monocyte- or lymphocyte-derived neutrophil-chemotactic and -activating factor/peptide, after stimulation with lipopolysaccharides (LPS), phorbol myristate acetate, IL-1, tumor necrosis factor-α (TNF-α), or typical T-lymphocyte stimulants [13–17]. Moreover, CXCL8 promoted rapid granulocytosis upon intravenous injection in rabbits [18]. Shortly thereafter, it was also discovered as a secreted protein from human fibroblasts or endothelial cells after stimulation with IL-1, TNF-α, LPS, or viral infection [19, 20]. Nowadays, CXCL8 is known to be produced and released by leukocytes and almost any other cell type in response to endogenous or exogenous pro-inflammatory stimuli [21].

### Structure of CXCL8

**Fig. 1:**
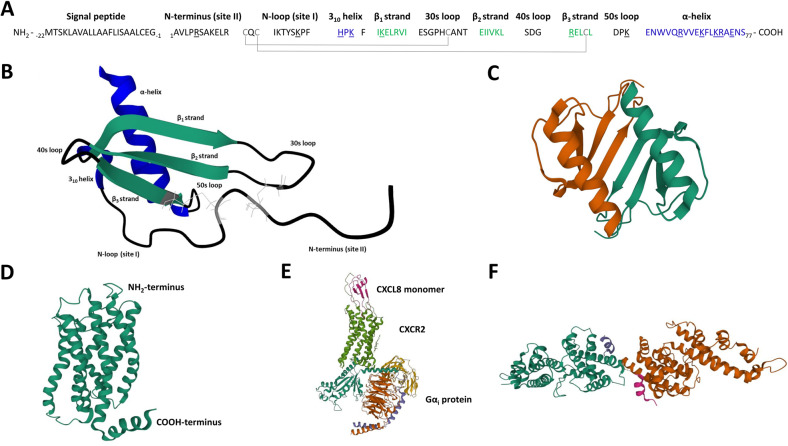
Primary sequence and 3D structures of CXCL8 and its receptors CXCR1, CXCR2, and ACKR1. **A** Primary sequence of human CXCL8 after translation, including the signal peptide and **B** 3D ribbon structure of the mature CXCL8 monomer as determined by NMR spectroscopy. All secondary structural elements are indicated in the amino acid sequence and the 3D structure. The four conserved cysteine residues and the disulfide bonds connecting them for stabilization of the CXCL8 structure are indicated in gray. The CXC motif can be found immediately COOH-terminally of the ELR motif. The three β-strands forming a β-sheet are colored in green. The COOH-terminal α-helix and the small 3_10_ helix are depicted in blue. The last two amino acids of the signal peptide (Glu-2 and Gly-1) can also be part of the mature CXCL8 protein due to alternative cleavage of the signal peptide. Underlined amino acid residues are implicated in GAG binding. **C** Dimer structure of CXCL8 in solution as determined by NMR spectroscopy. Monomer units are colored in orange and green. The CXCL8(6–77) 3D structure was drawn from PDB accession code 1IL8. **D** 3D structure of CXCR1 as determined by solid-state NMR spectroscopy and drawn from PDB accession code 2LN [34]. **E** Cryo-electron microscopy structure of monomeric CXCL8-activated human CXCR2 in complex with the Gα_i_ protein, drawn from PDB accession code 6LFO. A similar 3D structure of dimeric CXCL8 activating CXCR2 can be found with PDB accession code 6LFM [35]. **F** X-ray diffraction heterotetramer structure of two NH_2_-terminal ectodomains (forming a helix) of ACKR1 (DARC) binding each to two molecules of the receptor binding domain of *Plasmodium vivax* Duffy binding protein (DBP-RII), drawn from PDB accession code 4NUV. DBP-RII monomers are indicated in orange and green. DARC monomers are depicted in purple and blue. A DBP-RII∶DARC heterotrimer structure, where a single ACKR1 ectodomain binds two DBP-RIIs, can be found with PDB accession code 4NUU [40]

The genomic structure of the *CXCL8* gene was determined in 1989 [22]. *CXCL8* is composed of 4 exons and 3 introns and is located on chromosome 4 locus q12-q21 in a region where other genes coding for CXCL8-related chemokines reside [23]. After transcription and translation, a monomer precursor protein of 99 amino acid residues is generated (Fig. 1A). In the endoplasmic reticulum, the 22 amino acid long NH_2_-terminal signal peptide is removed generating a 77 amino acid long mature protein CXCL8(1–77), which is secreted by the cells. Alternative, but less common, signal peptide cleavage generates a 79 amino acid CXCL8(−2–77) protein [24]. CXCL8 can reversibly exist as a monomer or a dimer [25]. The CXCL8 monomer (Fig. 1A, B) possesses an unstructured, flexible, NH_2_-terminal domain (N-terminus) preceding the first two cysteine residues which are followed by an extended irregular N-loop. This loop is pursued by a small 3_10_ helix and three antiparallel β-strands connected by turns known as the 30s-, 40s-, and 50s-loop, which reflects the numbering of residues in the mature protein. Finally, a COOH-terminal α-helix is formed. CXCL8 is an ELR^+^ CXC chemokine, since the first two NH_2_-terminal cysteine residues (Cys12 and Cys14) following the ELR motif are separated by one residue (Gln13). The disulfide bridges between Cys12 and Cys39 (in the 30 s loop) and Cys14 and Cys55 (in the third β-strand) are important to maintain structural integrity [26, 27]. CXCL8 homodimers are mainly formed by hydrogen bonds between residues in the first β-strand of each subunit and are further stabilized by interactions between the ends of the COOH-terminal α-helices with the β-sheet of the opposing subunit [28]. The three-dimensional structure of the CXCL8 dimer in solution, as determined by nuclear magnetic resonance (NMR) spectroscopy and X-ray crystallography in 1990–1991, consequently comprises two symmetry-related antiparallel α-helices that rest on top of a six-stranded antiparallel β-sheet derived from two three-stranded Greek keys, one from each monomer unit (Fig. 1C) [29, 30].

### Receptors for CXCL8

The first isolation of a cDNA encoding a human CXCL8 receptor was published in 1991 [31, 32], and analysis of the putative structure revealed it was most closely resembling to formyl peptide GPCRs. CXCL8 interacts with the chemokine receptors CXCR1 (in old nomenclature also IL-8RA or IL-8R1) and CXCR2 (IL-8RB or IL-8R2) and with the atypical chemokine receptor ACKR1, also called Duffy antigen/receptor for chemokines (DARC) [3].

CXCR1 and CXCR2 are encoded by the single copy genes *CXCR1* and *CXCR2* located on chromosome 2q34-q35 and share high sequence homology [33]. A three-dimensional structure of the CXCR1 protein was first solved by solid-state NMR spectroscopy in 2012 (Fig. 1D) [34]. Recently, also the first cryo-electron microscopy structures of both dimeric and monomeric CXCL8-activated human CXCR2 in complex with the Gα_i_ subunit of the heterotrimeric GTP binding protein (G protein) were obtained (Fig. 1E) [35]. Indeed, CXCR1 and CXCR2 are rhodopsin-like class A GPCRs, composed of seven transmembrane domains connected by three extra- and intracellular loops. Due to the presence of a DRYLAIV motif within the second intracellular loop at the end of transmembrane domain 3, CXCR1 and CXCR2 induce downstream signaling via G proteins [3]. Both receptors are predominantly expressed on neutrophils but can also appear on IL-13- and IL-14-stimulated monocytes, T-lymphocytes, dendritic cells, mast cells, basophils, eosinophils, natural killer cells, myeloid-derived suppressor cells (MDSCs), and non-leukocytes like keratinocytes, fibroblasts, neurons, astrocytes, endothelial cells, epithelial cells, smooth muscle cells, hepatocytes, and melanocytes [3, 21]. CXCR1 is bound with high affinity and activated by the human chemokines CXCL6/GCP-2 and CXCL8 and weakly by CXCL5/epithelial-derived neutrophil-activating peptide 78 (ENA-78). However, upon appropriate NH_2_-terminal truncation, CXCL5 becomes a high affinity CXCR1 ligand as well [36]. CXCR2 is activated by all ELR^+^ CXC chemokines (CXCL1 to 3 and CXCL5 to 8) [3]. CXCR1 and CXCR2 can appear as monomers on cell membranes, but also form homodimers and heterodimers with each other. These interactions seem to be regulated by receptor expression levels and ligand activation and can contribute to receptor assembly and trafficking to the cell membrane [37]. Moreover, CXCR2 can form heterodimers with the seven-transmembrane domain receptor CCRL2, which is typically upregulated during inflammation and can fine-tune CXCR2-mediated neutrophil recruitment by promoting its expression and function. Finally, interaction of CXCR2 with opioid receptors and a glutamate receptor was described. Further detailed information can be found in the review from D’Agostino et al. [38].

**Fig. 2:**
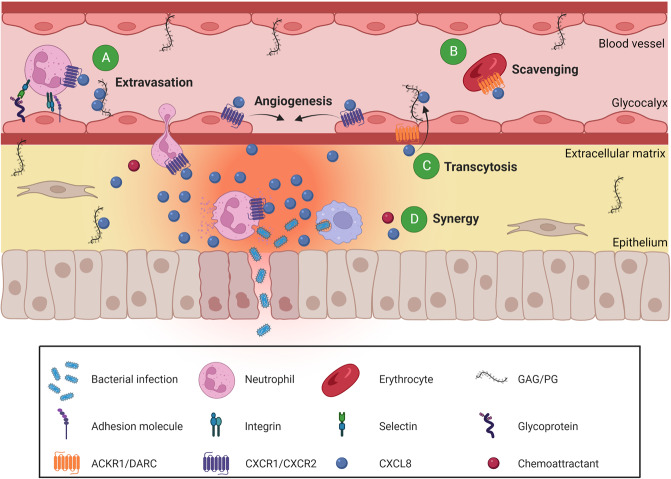
CXCL8-mediated neutrophil attraction to and activation at the inflammatory site. Upon an inflammatory trigger such as bacterial infection, several tissue cells and tissue-resident leukocytes produce CXCL8. CXCL8 establishes a concentration gradient from the production site to the blood vessels guiding neutrophils toward the inflammatory site, where they eliminate the pathogens and resolve acute inflammation. Afterwards, CXCL8 may promote angiogenesis by stimulating endothelial cell proliferation and migration to repair the damaged tissue. CXCL8 activity is regulated by: **A** The need for immobilization of CXCL8 on endothelial GAGs inhibiting proteolytic degradation and diffusion in the blood stream, establishing and maintaining a concentration gradient toward the inflammatory site. **B** Removal of non-immobilized CXCL8 from the bloodstream by binding to ACKR1 expressed on erythrocytes, preventing systemic leukocyte activation and providing a chemokine reservoir. **C** Translocation of CXCL8 from the extracellular matrix to the surface of the endothelial layer (transcytosis), which is controlled by binding to GAGs and endothelial ACKR1. **D** Synergy of CXCL8 with other chemoattractants to amplify the inflammatory response

The atypical chemokine receptor ACKR1 is expressed on post-capillary venular endothelial cells, Purkinje neurons of the cerebellum, erythrocytes (where it was discovered as the Duffy blood group antigen), but not on leukocytes [3, 39]. ACKR1 is also used by *Plasmodium vivax* and *Plasmodium knowlesi* as an entry receptor for erythrocyte invasion (Fig. 1F). A remarkable resistance to these malaria parasites is found in the majority of the Sub-Saharan African population due to a silencing mutation in the promoter region of *ACKR1*, located on chromosome 1q23.2, abolishing ACKR1 expression on erythrocytes but retaining expression on endothelial cells (Duffy-negative phenotype) [40, 41]. Moreover, ACKR1-expressing nucleated erythroid cells are described to contribute to the regulation of hematopoiesis in the bone marrow, establishing a link between neutropenia and a specific *ACKR1* gene variant causing the Duffy-negative phenotype [42]. Furthermore, ACKR1 binds over 20 different CC and CXC chemokines. The receptor is structurally composed of seven transmembrane domains but lacks a DRYLAIV motif and therefore does not activate G proteins. Instead, ACKR1 functions as a scavenging “sink” receptor on erythrocytes, retaining bound chemokines and thereby regulating their bioavailability in circulation (Fig. 2). As such, ACKR1 prevents excessive circulating chemokine concentrations and systemic leukocyte stimulation but also provides a blood reservoir (buffer) of chemokines, extending their half-life [3, 43]. On the other hand, ACKR1 promotes inflammation by binding, internalization, and transport of CXCL8 and other chemokines from the basolateral toward the apical side of endothelial cells, which facilitates immobilization and presentation to leukocytes promoting their extravasation [44]. GAGs probably participate in this chemokine transcytosis process as well (Fig. 2) [45, 46]. Finally, ACKR1 expression in endothelial junctions could promote the neutrophil transendothelial migration itself, as it interacted with CXCL2 creating a junctional chemokine “depot” in mice [47].

### Functions of CXCL8

Neutrophil recruitment to inflammatory sites is mediated by multiple chemoattractant subfamilies: chemotactic lipids (e.g. leukotriene B4), complement anaphylatoxins C3a and C5a, N-formylated peptides, and chemokines [48, 49]. CXCL1 to 3 and CXCL5 to 8 are the seven human neutrophil-attracting and -activating chemokines, with CXCL8 as the most potent and often abundantly produced protein. Extravasation of neutrophils from blood to the inflammatory site happens in a coordinated process. It comprises neutrophil rolling over the activated endothelium, followed by ligand-activation of chemoattractant receptors leading to integrin activation and a consequent tight endothelial adhesion. Thereafter, neutrophils transmigrate from the bloodstream through the endothelium and move further toward high concentrations of chemoattractants in the inflamed tissue (Fig. 2) [49]. Essential for efficient inflammatory neutrophil recruitment in vivo is the immobilization of CXCL8 on GAGs/proteoglycans and the interaction with CXCR1 and CXCR2 to induce (trans)migration [46, 50]. Interestingly, murine neutrophils can also exert reverse transendothelial migration from inflamed tissues to the circulation, which has been specifically linked to aging. This process was regulated by increased ACKR1 retention of mast cell-derived CXCL1 at endothelial cell junctions in aged venules, inducing neutrophil internalization of CXCR2 and the reverse transendothelial migration process. Eventually, this led to remote organ damage established by the re-circulating neutrophils [51]. Since mice possess only three CXCR2 ligands and no CXCL8 (*vide infra*), it is not evident to translate these data to the human system.

Apart from regulating neutrophil recruitment and activation, in specific conditions CXCL8 is described to regulate endothelial adhesion, chemotaxis, and activation of other leukocytes, including IL-13/IL-4-stimulated monocytes [52], subsets of CD8^+^ T-lymphocytes [53], and mast cells [54]. Moreover, CXCL8 can regulate non-immune cell migration and stimulate corneal neovascularization in vivo [55]. Indeed, CXCL8 promotes angiogenesis like all ELR^+^ chemokines [56]. As such, CXCL8 promotes tissue repair, blood flow restoration after organ transplantation, and wound healing. The production of CXCL8 and other neutrophil-attracting chemokines by wounded epithelial cells and platelets first supports neutrophil migration to the wound area for pathogen elimination and removal of cell debris. Thereafter, CXCL8 promotes angiogenesis by stimulating migration and proliferation of CXCR2-expressing neovascularizing endothelial cells, forming new blood vessels (Fig. 2). Finally, CXCL8 supports re-epithelialization by stimulating keratinocyte proliferation [57].

### Interaction of CXCL8 with GAGs, receptors & other proteins

#### CXCL8 binding to GAGs

CXCL8 binding to GAGs present on the endothelium, in the basement membrane, or extracellular matrix prevents diffusion and establishes/maintains a concentration gradient for neutrophil chemotaxis toward the inflammatory site (Fig. 2) [5, 58]. CXCL8 mainly uses its COOH-terminal α-helix to bind to GAGs [50], but also the proximal loop around residues 18–25 seems to play a role. More specifically, the basic residues Lys20, His23, Lys25, and Lys28 in the N-loop, 3_10_ helix, and first β-strand and Arg52, Lys59, Arg65, Lys69, Lys72, Arg73, and Glu75 located in the COOH-terminal part (mainly the COOH-terminal α-helix) of CXCL8(1–77) were confirmed to be important for GAG binding [4]. Moreover, citrullination of CXCL8 on Arg5 reduces its affinity for heparin and heparan sulfate (*vide infra*) [59], suggesting that this residue can be involved in GAG binding (Fig. 1A). The exact CXCL8 binding site on GAGs is not completely elucidated and may even differ depending on the GAG structure. It was found that CXCL8 mainly binds as a dimer by strong ionic interactions (affinity in the micromolar range) between basic residues of CXCL8 and two sulphated domains (of approximately 6 monosaccharide units separated by a region of maximally 14 monosaccharide residues) of the GAG heparan sulfate [60]. More specifically, the 6-O-sulfate groups in chondroitin-6-sulfate, heparin, and heparan sulfate are important for the interaction with CXCL8. Indeed, CXCL8 shows some selectivity toward specific types of GAGs present in the extracellular matrix, having strongest interactions with (subfractions of) heparin, followed by heparan sulfate, chondroitin sulfate, hyaluronic acid, and dermatan sulfate [4]. Interestingly, GAG binding not only immobilizes chemokines but can protect them, at least partially, from proteolysis. As proteases drastically alter the biological activity of CXCL8 (*vide infra*), this could further regulate the intensity of the inflammatory response. Protection from proteolytic cleavage by GAGs could be a result of inaccessibility of the active site from the protease for the chemokine or stabilization of oligomerized chemokine structures hiding the cleavage site for the protease [61].

#### CXCL8 binding to its receptors

##### Interactions with CXCR1 & CXCR2

CXCL8 reversibly exists as a monomer or a dimer but it is not completely understood whether (a) CXCL8 monomers or dimers eventually activate their receptors and (b) how the monomer-dimer equilibrium determines CXCL8 gradient formation and neutrophil recruitment in the presence of GAGs. By in vitro cellular studies, it was shown that CXCL8 may bind and activate its receptor as a monomer [62] or a dimer [63]. Later, the CXCL8 monomer was demonstrated to be more potent than the dimer in receptor binding and neutrophil activation experiments in vitro, which was mainly achieved by an increased activity response after binding to CXCR1, and not CXCR2 [64]. In the in vivo setting, both monomeric and dimeric CXCL8 were functional for neutrophil recruitment to the lungs and the peritoneum [65]. Although GAG interactions are essential for the in vivo chemotactic activity of CXCL8, it is not completely elucidated if immobilized or soluble CXCL8 eventually activates CXCR1 and CXCR2. Due to an overlap between GAG-binding and GPCR-binding sites in the NH_2_-terminus of CXCL8, it is less likely that a monomer of CXCL8 can simultaneously interact with both GAGs and receptors [61]. Indeed, using solution NMR spectroscopy, it was shown that although both CXCL8 monomers and dimers can bind to GAGs and receptors, heparin-bound CXCL8 monomer or dimer cannot simultaneously bind CXCR1 or CXCR2 and activate neutrophils. When a CXCL8 dimer is bound to a single GAG chain, one could however expect that the first monomer can bind the GAG while the second monomer binds the receptor (bridge model), but no experimental evidence is found for this theory. Consequently, as the CXCL8 monomer is the high-affinity ligand for the receptors, it was suggested that the free, soluble monomer eventually binds and activates the receptors inducing neutrophil recruitment to inflamed tissues. However, the levels of soluble CXCL8 in the glycocalyx would still be dictated by local GAG interactions with CXCL8 (predominantly CXCL8 dimers, as they bind GAGs with higher affinity than monomers). Indeed, this large GAG-bound CXCL8 reservoir could be in equilibrium with the free chemokine form which ensures a constant chemokine replenishment for receptor binding and may delay diffusion of soluble CXCL8 in the blood stream due to steric hindrance and transient GAG interactions (Fig. 2) [66, 67]. This concept follows the recently proposed “chemokine cloud” model, where GAGs provide an immobilized chemokine depot, releasing and maintaining a cloud of chemokines in solution sequestered within the glycocalyx, with these soluble chemokines eventually interacting with the leukocyte receptors [6].

Within CXCL8, the unstructured NH_2_-terminal part is the site for CXCR1/CXCR2 binding and activation [68]. Indeed, according to the two-step/two-site binding mechanism of chemokines to chemokine receptors, the NH_2_-terminal N-loop (core globular domain) of CXCL8 first interacts with the NH_2_-terminus of the receptor (defined as chemokine recognition site I) providing affinity and specificity. Afterwards, the CXCL8 NH_2_-terminus binds within the pocket of the chemokine receptor where it interacts with the receptor transmembrane and extracellular loop residues (defined as chemokine recognition site II) leading to receptor activation (Fig. 1A) [26, 61, 69]. However, several studies indicate that this model is probably too simplified, in a way that both ligand-binding sites are probably linked to each other rather than being completely independent and that additional interactions between the chemokine and its receptors are required [26, 69].

##### CXCL8-induced signaling

**Fig. 3:**
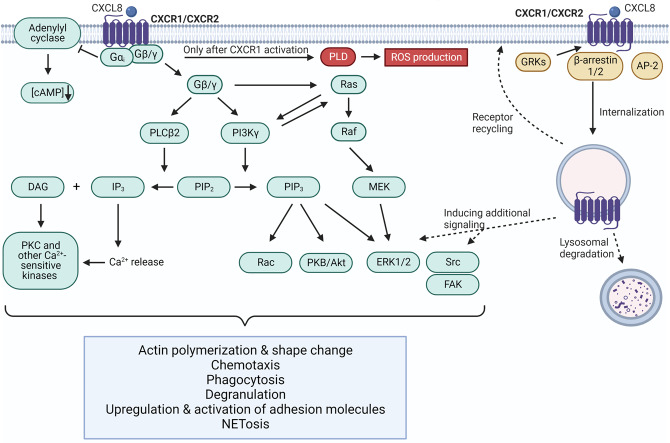
CXCL8 binding to CXCR1 and CXCR2 on neutrophils activates G protein- and β-arrestin-mediated signal transduction pathways. G protein-mediated signaling activates MAPK, PLC, and PI3K pathways leading to neutrophil chemotaxis and effector functions. ROS production was suggested to be specifically linked to activation of PLD after CXCR1, but not CXCR2, activation since CXCL8, but not CXCL1, induced ROS [72]. CXCL8 binding to CXCR1/CXCR2 also leads to desensitization of G protein-mediated signaling, through internalization of the receptors mainly mediated by β-arrestins. Afterwards, receptors can be degraded, recycled to the membrane, or induce an additional round of MAPK or tyrosine kinase signaling

CXCR1/CXCR2 activation on neutrophils is coupled to G protein and β-arrestin-mediated signal transduction cascades (Fig. 3). G protein-mediated signaling starts with binding of CXCL8 to the receptor inducing a conformational change. This leads to the exchange of guanosine diphosphate (GDP) for guanosine trisphosphate (GTP) and dissociation of the Gβ/γ subunit from the Gα subunit of the heterotrimeric G protein, which is bound to the 2^nd^ and 3^rd^ intracellular loop and the COOH-terminus of the receptor. For CXCR1- and CXCR2-mediated signaling, Gα is mostly of the inhibitory Gα_i_ type. Gα_i_ inhibits the function of adenylyl cyclase lowering levels of cyclic adenosine monophosphate (cAMP) which is generated from ATP. The Gβ/γ subunit activates phospholipase C (PLC)β2 initiating the conversion of phosphatidylinositol (4,5)-biphosphate (PIP_2_) into inositol 1,4,5-trisphosphate (IP_3_) and diacylglycerol (DAG). This results in release of Ca^2+^ from the endoplasmic reticulum into the cytosol and activation of Ca^2+^-sensitive protein kinases like protein kinase C (PKC), which is crucial for the initiation of neutrophil chemotaxis. Moreover, phosphoinositide 3-kinase (PI3K)γ will be activated by the Gβ/γ subunit, which converts PIP_2_ on his turn into phosphatidylinositol (3,4,5)-trisphosphate (PIP_3_) leading to downstream activation of the GTPase Rac, phosphokinase B (PKB or Akt), and extracellular signal-regulated kinases (ERK1/2). ERK1/2 may also be activated by the classical Ras-Raf-MEK-ERK signaling pathway, for which activation may be dependent on PI3Kγ activity. However, no consensus is reached with some conflicting publications claiming that the MEK/ERK activation may or may not be crucial for induction of neutrophil chemotaxis. Finally, Src kinases and focal adhesion kinase (FAK) were shown to be activated via so far only partially characterized pathways induced by G proteins or β-arrestins (*vide infra*). Eventually, this G protein-mediated signaling induces neutrophil functions like actin polymerization (essential at the leading edge of the neutrophil to allow transmigration), upregulation and activation of adhesion molecules, chemotaxis, phagocytosis, reactive oxygen species (ROS) production, degranulation, and neutrophil extracellular trap (NET) formation [48, 70, 71]. The exact contribution of each signal transduction pathway to specific neutrophil functions is far from completely elucidated. However, some neutrophil effector functions are reported to be linked to activation of either CXCR1 or CXCR2. The production of ROS for microbial clearance and the activation of phospholipase D (PLD) were specifically associated with CXCL8-induced CXCR1 activation. Phosphatidic acid, which results from conversion of phosphatidylcholine by PLD, is known to activate the NADPH oxidase for superoxide anion production [72]. Besides, CXCR2 was suggested to be the main (but not exclusive) mediator for neutrophil chemotaxis [73], as it is more sensitive to low ligand concentrations and more quickly downregulated at the inflammatory site due to more rapid internalization compared to CXCR1 (*vide infra*) [26, 71].

Chemokine-induced G protein-coupled signaling needs to be strictly regulated and eventually terminated at the inflammatory site. This is to prevent constitutive signaling and ensure that chemokine receptors remain responsive for a renewed stimulation. Therefore, upon CXCL8 stimulation, CXCR1/CXCR2 will be rapidly desensitized (homologous desensitization), mediated by phosphorylation of serine and threonine residues at the COOH-terminus by intracellular G protein-coupled receptor kinases (GRKs). This enables binding of β-arrestin 1/2, which leads to uncoupling of G protein and conventional signaling, and dynamin- and clathrin-mediated receptor internalization and sequestration into endosomes. Internalization can also be mediated by the adaptor protein-2 (AP-2), which does not require phosphorylation of the receptor. Interestingly, CXCR2 can also be cross-phosphorylated and as such desensitized for CXCL8 by other chemoattractant receptors (heterologous desensitization) like formyl peptide receptor 1 (FPR1) and C5aR. After internalization into endosomes, the receptors can be degraded in lysosomes, recycled to the plasma membrane, or initiate a second round of signaling via the mitogen-activated protein kinase (MAPK) and tyrosine kinase (e.g. Src) pathways [26, 70, 71]. The structural basis for differential activation of G protein and β-arrestin signaling pathways is suggested to be mediated by the 30 s loop residues in CXCL8, functioning as a conformational switch coupling site I and site II interactions and controlling the distribution of conformational substates for G protein versus β-arrestin signaling pathways [26].

#### CXCL8 binding to other proteins

Besides homodimers, CXCL8 is described to form heterodimers. CXCL8 can interact with the platelet chemokine CXCL4/platelet factor 4 (PF-4), after incubation of CXCL4 tetramers together with CXCL8 dimers. This heterodimerization increased the anti-proliferative activity of CXCL4 on endothelial cells as well as the chemotactic effect of CXCL8 on CXCR2-transfected cells [74]. Moreover, the CXCL8-CXCL4 interaction inhibited the CXCL8-mediated metabolic activation of CD34^+^ hematopoietic progenitor cells [75]. CXCL8 can also directly bind to TNF-stimulated gene/protein-6 (TSG-6), a multifunctional protein expressed by neutrophils, monocytes, and endothelial cells in response to pro-inflammatory cytokines. Due to binding to the GAG binding site of CXCL8 and the GAG itself, TSG-6 prevents CXCL8-GAG interactions and thereby inhibits CXCL8-induced neutrophil migration. As such, it protected tissues from acute inflammation [76, 77].

### Regulation of CXCL8 activity

**Fig. 4:**
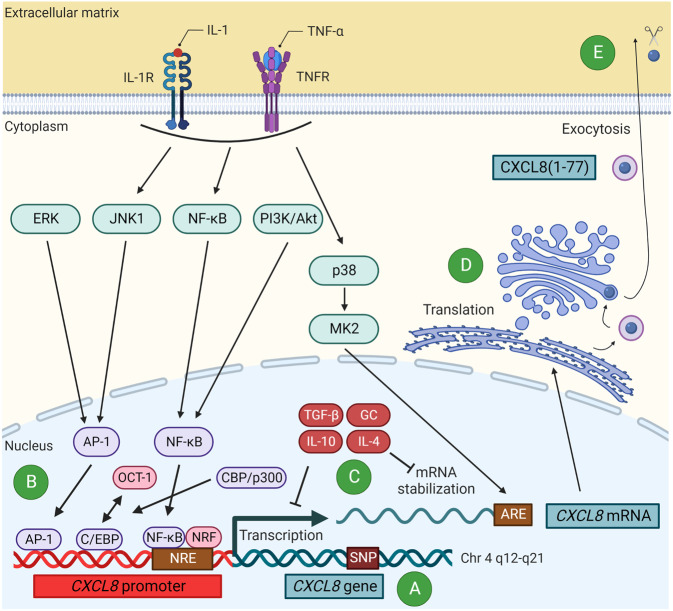
The production process of CXCL8 after inflammatory stimulation is tightly regulated. In response to an inflammatory trigger, pro-inflammatory cytokines like IL-1 or TNF-α induce the production of CXCL8 by stimulation of their receptors. This induces downstream signaling pathways resulting in activation of NF-κB and the AP-1 complex, which translocate into the nucleus and initiate transcription of the *CXCL8* gene and production and release of CXCL8. This process is regulated at different levels. **A** Specific *CXCL8* polymorphisms influence the CXCL8 production levels. **B** Transcription is only initiated after de-repression of the *CXCL8* gene promoter, mediated through association of NF-κB with NF-κB-repressing factor (NRF) and the negative regulatory element (NRE) in the promoter and replacing octamer-1 (OCT-1) by the transcription factor CCAAT/enhancer-binding protein (C/EBP). This recruits the co-activator CREB-binding protein (CBP)/p300 which results in histone hyperacetylation and chromatin remodeling so that AP-1 and NF-κB can activate the gene transcription process. Anti-inflammatory stimuli like IL-10 and TGF-β can block this transcription process. **C** After production, the labile *CXCL8* mRNA needs to be stabilized by a MAP kinase-activated protein kinase 2 (MK2)-dependent AU-rich cis-elements (ARE)-targeted mechanism through activation of the p38 MAPK pathway. This stabilization is promoted by LPS, IL-1, TNF-α, IFN-γ, nitric oxide, and hypoxia (not shown) and repressed by IL-4, IL-10, and glucocorticoids (GC) promoting mRNA degradation. **D** After mRNA translation, CXCL8 localizes intracellularly in the Golgi apparatus, from where it is secreted (constitutive secretory pathway). **E** After exocytosis, CXCL8 can be subjected to multiple post-translational modifications like proteolysis with profound effects on its activity

**Fig. 5:**
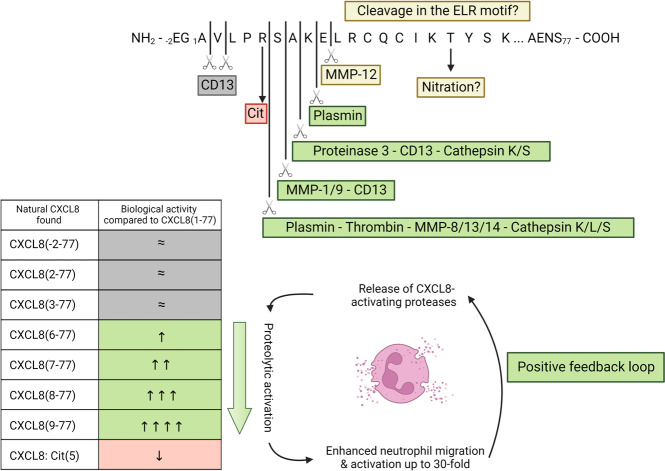
Post-translational modifications affect the biological activity of CXCL8. Natural CXCL8 has been identified as a partially citrullinated protein and as multiple NH_2_-terminally truncated proteoforms (green, red, and gray boxes). Citrullination of CXCL8 on Arg5 significantly reduces its biological activity whereas removal of 5–8 amino acids significantly potentiates the activity of CXCL8 up to almost 30-fold, compared to CXCL8(1–77). Moreover, due to induction of increased neutrophil migration and activation, additional proteases are released which again enhance the proteolytic activation of CXCL8 (positive feedback loop). Further proteolytic cleavage in the ELR motif and nitration have not been reported on natural CXCL8 (yellow boxes) but CXCL8(10–77) could be generated after incubation of CXCL8 with MMP-12. Cleavage in the ELR motif or nitration reduce or abolish the activity of CXCL8 in vitro

The production, release, and activity of CXCL8 and other chemokines is strictly regulated at multiple levels. This is required to prevent unlimited or inappropriate leukocyte attraction and is probably also important for resolution of inflammation (Figs. 2, 4 and 5) [78].

#### Gene level

Within the *CXCL8* gene, several single nucleotide polymorphisms (SNPs) are described, which may have a profound effect on the level of protein synthesis (Fig. 4). These SNPs are associated with cancer and airway, gastrointestinal, neurological, and specific diseases like systemic lupus erythematosus (SLE) [79]. One specific SNP (IL-8-845C) in the promoter region of *CXCL8* might predispose African Americans to more severe SLE nephritis [80]. Moreover, in the *CXCL8, CXCR1*, and *CXCR2* genes, specific SNPs are associated with increased risk for colon or rectal cancer [81].

#### Transcriptional level

The expression of the *CXCL8* gene is upregulated locally after receptor activation by pro-inflammatory stimuli like IL-1 or TNF-α in response to an inflammatory trigger. Transcription is then initiated by the coordinate action of different signal transduction pathways (Fig. 4) [71, 82–84]. These include nuclear factor kappa-light-chain-enhancer of activated B cells (NF-κB) and c-Jun N-terminal kinase 1 (JNK1) signaling pathways, which result in activation of NF-κB and activator protein-1 (AP-1) complex (composed of c-Fos and c-Jun). Moreover, PI3K/Akt and ERK signaling can also mediate activation of NF-κB and AP-1, respectively. These transcription factors then translocate into the nucleus and jointly promote the transcription of the *CXCL8* gene. For the transcription process itself, the *CXCL8* promoter needs to be de-repressed. On the one hand, this is mediated by associating NF-κB with NF-κB-repressing factor (NRF) and the negative regulatory element (NRE) in the *CXCL8* promoter, so that NRF becomes a co-activator. On the other hand, octamer-1 (OCT-1) is replaced by the transcription factor CCAAT/enhancer-binding protein (C/EBP). C/EBP, once bound to the promoter, recruits the co-activator CREB-binding protein (CBP)/p300 which results in histone hyperacetylation and remodeling of the chromatin. Consequently, AP-1 and NF-κB will activate the *CXCL8* transcription process. A third signaling pathway activated by pro-inflammatory stimuli, the p38 MAPK pathway, stimulates MAP kinase-activated protein kinase 2 (MK2). This is important for stabilization of the labile *CXCL8* mRNA contributing to upregulation of CXCL8 production (Fig. 4). In contrast, endogenous anti-inflammatory cytokines like IL-10 and transforming growth factor β (TGF-β) can block the transcription of pro-inflammatory chemokine genes. IL-10 for instance inhibits NF-κB activation in human monocytes, thereby impeding LPS-stimulated production of CXCL8 [85].

#### Post-transcriptional level

*CXCL8* mRNA can be rapidly degraded by instability due to the presence of AU-rich cis-elements (ARE) in the 3’ untranslated region. However, as previously mentioned, the p38 MAPK pathway stabilizes the mRNA through a MK2-dependent, ARE-targeted mechanism [84]. LPS, IL-1, TNF-α, IFN-γ, nitric oxide, and hypoxia promote mRNA stabilization (mainly through the p38 MAPK pathway) and therefore upregulate CXCL8 production, whereas IL-4, IL-10, and glucocorticoids like dexamethasone and hydrocortisone promote degradation or decrease the stability of *CXCL8* mRNA (Fig. 4) [85, 86]. Furthermore, small non-coding microribonucleic acids (miRNAs) can directly or indirectly up- or downregulate CXCL8 production by causing degradation and/or translational repression of mRNA. So it was demonstrated that miR-155, abundantly expressed in the lungs of cystic fibrosis patients, indirectly promotes inflammation by driving hyperexpression of CXCL8, but that miR-17 overexpression in cystic fibrosis airway epithelial cells decreases CXCL8 production [71].

#### Translational level

After nuclear export and ribosomal translation of the *CXCL8* mRNA, the CXCL8 protein first localizes intracellularly in the Golgi apparatus, from where it is secreted through the constitutive secretory pathway (Fig. 4). Interestingly, after prolonged stimulation (for hours) with pro-inflammatory mediators like IL-1β, CXCL8 can be stored in intracellular secretory storage granules, more specifically in Weibel-Palade bodies from microvascular endothelial cells. After a period of discontinued stimulation, additional re-stimulation with e.g. histamine, thrombin, or fibrin, can induce rapid release of CXCL8 from these bodies without requirement of de novo protein synthesis. As such, the storage of CXCL8 in these bodies may serve as endothelial cell “memory” for a preceding inflammatory insult [87, 88].

#### Post-translational level

After synthesis, chemokines can be subjected to chemical and enzymatic post-translational modifications like proteolysis, glycosylation, nitration, or citrullination. These modifications are mediated by multiple enzymes and other protein-modifying agents, which are upregulated and/or released during inflammatory conditions and can lead to altered chemokine activity or chemokine receptor specificity [78, 89]. Natural CXCL8 is originally found in supernatants of stimulated cells as multiple NH_2_-terminally truncated proteoforms with a loss of up to eight amino acid residues [90]. Intact CXCL8(1–77) and truncated CXCL8(6–77) were the most abundantly found natural proteoforms, with the former being the main proteoform derived from stimulated endothelial cells and fibroblasts and the latter from T-lymphocytes and monocytes [19, 91, 92]. CXCL8 proteoforms are characterized by profound differences in their neutrophil chemotactic and functional activity up to almost 30-fold. An overview of the different post-translational modifications with their characteristic effects on the biological activity of CXCL8 is provided in Table 1 and Fig. 5. A summary is provided below.

**Table 1:** Overview of post-translational modifications influencing the biological activity of CXCL8 [24, 59, 68, 92, 93, 95–97, 182, 183, 296–300]

Colored rows indicate CXCL8 proteoforms that have been naturally found in biological samples. Gray rows = comparable activity, green rows = increased activity & red rows = decreased activity. All comparisons are made with intact CXCL8(1–77), unless otherwise indicated. This table is partially adapted from Vanheule et al. [89]

*ACKR1* atypical chemokine receptor 1, *GAG* glycosaminoglycans, *MMP* matrix metalloproteinase

Alternative cleavage of the signal peptide or minor proteolytic truncation of one or two NH_2_-terminal amino acids of intact CXCL8(1–77) do not significantly alter the biological activity of the chemokine [24]. However, proteolytic removal of five up to eight NH_2_-terminal residues significantly increases the CXCL8 activity. Resulting proteoforms are highly potent neutrophil attractants and activators, with an apparent progressive increase in activity upon further truncation. CXCL8(9–77) may be the most potent neutrophil-attracting CXCL8 proteoform in vivo, at least in mice upon binding to murine receptors (Table 1) [93]. Moreover, neutrophils probably enhance their own recruitment and activation by secreting proteases that process CXCL8 into more active proteoforms initiating a positive feedback (auto-amplification) loop (Fig. 5). Further NH_2_-terminally truncated proteoforms, cleaved in or beyond the ELR motif, have not been found yet in biological samples. As the ELR motif is essential for receptor binding and signaling activity [94], chemically synthesized CXCL8 proteoforms cleaved in this motif typically had significantly impaired biological activity in vitro (Table 1) [68]. Interestingly however, the proteoform CXCL8(10–77), which was generated by incubation with the macrophage metalloelastase MMP-12 [95] and may also result from further aminopeptidase-dependent truncation of CXCL8(9–77), had some remaining affinity for the neutrophil receptors and no impaired chemotactic potency in vitro. Nevertheless, CXCL8(10–77) induced a drastically lower release of neutrophil elastase compared to stimulation with CXCL8(1–77) [68]. Besides proteolysis, CXCL8 can be citrullinated; a post-translational modification mediated by peptidylarginine deiminases which modify an arginine to a citrulline residue. Citrullination of Arg5 in CXCL8, generating CXCL8(1–77)Cit(5), was discovered on 14% of natural leukocyte-derived CXCL8(1–77) and mainly seems to dampen tissue inflammation mediated by CXCL8 [59]. In rabbit blood however, citrullinated CXCL8 enhanced neutrophil mobilization after intravenous injection and had a reduced capacity to bind ACKR1, which might delay its clearance from the circulation (Table 1) [96]. A final post-translational modification of CXCL8 is nitration. Nitration (on Tyr18) by peroxynitrite reduced the ability of CXCL8 to bind neutrophils and its neutrophil chemotactic activity in vitro [97]. However, peroxynitrite (which is formed by reaction between nitric oxide and superoxide) has a short half-life and therefore no in vivo evidence for nitration has been provided so far.

We recently developed a method that can be exploited to detect and quantify intact CXCL8(1–77), elongated CXCL8(−2–77), and all naturally occurring truncated and activated CXCL8 proteoforms in biological (patient) samples, known as “immunosorbent nano-scale liquid chromatography tandem mass spectrometry proteoform analysis (ISTAMPA)”. Such a method is essential, as the proteoforms cannot be distinguished by standard immunoassays like ELISAs or western blot since (a) available antibodies fail to discriminate between the proteoforms and (b) western blot cannot detect minor differences in molecular mass due to the subtle proteolysis. With this technique, all the natural CXCL8 proteoforms were found in the synovial fluids of arthritic patients [98]. Since proteases truncating CXCL8 are usually upregulated in inflammatory conditions, detecting different CXCL8 proteoforms with highly differential activity in samples from patients with inflammatory diseases will be relevant. Indeed, CXCL8 proteoforms with high activity may significantly enhance the recruitment and activation of neutrophils amplifying inflammation. This could allow future identification of therapeutic targets e.g. by inhibiting the proteolytic activation of CXCL8 toward specific CXCL8 proteoforms with high activity.

#### Functional level

After synthesis and post-translational modifications, interactions with GAGs, ACKR1, and synergy with other chemokines further regulate the activity of CXCL8. Firstly, GAGs not only immobilize CXCL8 for neutrophil (trans)migration (*vide supra*) but are also described to influence CXCL8-mediated neutrophil functions. GAGs enhanced the CXCL8-induced ROS production [99] but inhibited elastase release from stimulated neutrophils [50], which could be a way to partially protect the tissue microenvironment from damage by lytic enzymes released from the migrating cells. Effects on in vitro neutrophil migration differed between specific GAGs; with heparan sulfate enhancing in vitro chemotaxis of neutrophils toward CXCL8 up to 4-fold [50], whereas heparin did not influence chemotaxis or even decreased it [50, 99]. Secondly, ACKR1 regulates the bioavailability of CXCL8 by binding to circulating erythrocytes but also translocates CXCL8 to the endothelial surface from the extracellular matrix (*vide supra*). Finally, CXCL8 can synergize with other chemokines (Fig. 2). This means that leukocyte recruitment and the inflammatory response induced by synergizing chemoattractants is higher than the cumulative effect of the individual chemoattractants. CXCL8 synergizes with several chemokines in vitro, as the chemotactic response of neutrophils toward a suboptimal concentration of CXCL8 was empowered by the chemokines CCL2/monocyte chemotactic protein 1 (MCP-1), CCL7/monocyte chemotactic protein 3 (MCP-3), CCL8/monocyte chemotactic protein 2 (MCP-2), and the constitutive chemokines regakine-1 and CXCL12, which are weak neutrophil attractants by themselves. For the latter, the synergistic effect could be reduced by plerixafor (AMD3100), a specific CXCR4 inhibitor, demonstrating the requirement of CXCL12 binding to its own receptor to obtain the synergistic effect [100]. The cellular mechanisms involved in this synergy are still debated but might be due to cooperation in signaling after chemokine and/or receptor heterodimerization [101]. In vivo, synergy in the recruitment of neutrophils to inflamed peritoneum was seen between CCL7 or CXCL12 and murine GCP-2, the most potent neutrophil-attracting chemokine in mice and likely functional homolog of human CXCL8 since it activates both CXCR1 and CXCR2 (*vide infra*) [102, 103].

### CXCL8 in diseases

Elevated levels of CXCL8 are found in inflamed tissue and the blood circulation in several inflammatory diseases [21, 71]. Here, we provide an update on the role of CXCL8 in some major disease categories (Table 2).

**Table 2:** Role of the CXCL8-CXCR1/CXCR2 axis in diseases

Disease		Effects of CXCL8-CXCR1/CXCR2	Ref.
Cancer		Promoting tumor survival, growth & migration Stimulating neovascularization in the tumor Inducing epithelial-to-mesenchymal transition, promoting invasion & metastasis Recruiting tumor-associated neutrophils (anti-tumor N1 & pro-tumor N2) Recruiting CXCR1- or CXCR2-expressing MDSCs Stimulating NETs release, which can coat tumor cells shielding them from immune cytotoxicity Recruiting tumor-associated macrophages & inducing an immunosuppressive M2 phenotype Promoting recruitment & proliferation of CXCR1/CXCR2-expressing cancer stem cells Inducing resistance to anti-tumor therapy Some rare studies indicate an anti-tumor effect of CXCL8	[57] [57] [104, 105] [107] [108, 109] [110] [111] [71, 105] [112, 113] [118, 119]
Cardiovascular diseases	Atherosclerosis	Inducing adhesion and chemotaxis of CXCR2-expressing foam cells & neutrophils Contributing to the early phase of atherosclerotic plaque formation Recruiting bone marrow endothelial progenitor cells promoting atherosclerotic plaque resolution	[21] [21] [120]
	Myocardial infarction	Promoting angiogenesis, neutrophil infiltration & infarction size	[121]
	Arterial hypertension	Promoting hypertension & vascular dysfunction by recruiting CXCR2^+^ pro-inflammatory cells	[122]
Pulmonary diseases	Acute lung injury/ARDS	Recruiting neutrophils, leading to neutrophil-associated damage Immune complexes of anti-CXCL8 autoantibodies suppress spontaneous neutrophil apoptosis	[21, 124] [123]
	Cystic fibrosis	Recruiting neutrophils CXCR1 expression on neutrophils is important for clearance of bacterial infections Promoting airway hyperresponsiveness by inducing contraction of airway smooth muscle cells	[125] [21, 126] [21]
	Asthma	Recruiting neutrophils Inducing bronchoconstriction by stimulating CXCR1/CXCR2-expressing airway smooth muscle cells Promoting angiogenesis of peribronchial blood vessels during allergic airway remodeling	[21] [21] [21]
	COPD	Recruiting neutrophils and CXCR2-expressing macrophages Neutrophilic proteases destroy tissue of the small airways	[125, 127] [128–130]
	IPF	Recruiting neutrophils Promoting angiogenesis Promoting collagen deposition & fibrosis	[133] [134, 135] [134]
Transplantation & IRI	Transplantation	Recruiting neutrophils leading to neutrophil-associated damage Rejection of transplanted organs	[136, 137] [136, 137]
	IRI	Recruiting neutrophils leading to neutrophil-associated damage	[138–140]
Arthritic diseases & pain	Arthritic diseases	Recruiting & activating neutrophils Promoting angiogenesis Maintaining chondrocyte phenotypic stability in chronic osteoarthritis	[142–144] [147] [153]
	Pain	Inducing sympathetic & neuropathic pain	[150–152]
Neurological diseases	Multiple sclerosis	Recruiting neutrophils Inducing blood-brain-barrier breakdown & development of autoimmune demyelination Uncertain effects on remyelination	[154, 156] [154, 156] [21, 154, 157]
	Alzheimer’s disease	Protecting human neurons from amyloid-β-induced neurotoxicity Inducing microgliosis and oxidative stress	[158] [159]
Kidney diseases & diabetes	Kidney diseases	Recruiting neutrophils & mediating damage to the kidneys	[21]
	Diabetes	Inducing pathogenesis of type 1 diabetes & diabetic kidney disease in type 2 diabetes	[160, 161]
Auto-immune diseases	Psoriasis	Autocrine stimulation of CXCR2-expressing keratinocytes in skin lesions Recruiting neutrophils Stimulating angiogenesis	[21] [21] [21]
	SLE	High levels & a *CXCL8* single nucleotide polymorphism are associated with severe disease	[80, 163]
Inflammatory bowel diseases		Recruiting & activating neutrophils and macrophages for clearance of pathogens Neutrophil-associated inflammatory damage Crosstalk between innate and adaptive immunity	[21] [21] [165]
Infectious diseases	General	Recruiting & activating neutrophils for clearance of pathogens Neutrophil-associated inflammatory damage	[21] [21]
	COVID-19	Increased activation state of circulating blood neutrophils Hyperactivated neutrophils in the lungs with severe proteolytic activity Pro-thrombotic neutrophils, characterized by degranulation and NET formation Anti-CXCL8 autoantibodies may reduce the severe systemic inflammation	[166] [168] [169] [170]
	Sepsis	Recruiting neutrophils & releasing NETs inducing bacterial clearance & tissue damage CXCR2 desensitization impairs adequate control of microbial dissemination Phospholipase D2 (PLD2) downregulates CXCR2 and inhibits NET release driving mortality	[21, 171] [21] [172]

*ARDS* acute respiratory distress syndrome, *COPD* chronic obstructive pulmonary disease, *IPF* idiopathic pulmonary fibrosis, *IRI* ischemia-reperfusion injury, *MDSC* myeloid-derived suppressor cell, *NET* neutrophil extracellular trap, *SLE* systemic lupus erythematosus

#### Cancer

The angiogenic and inflammatory effect of CXCL8 promotes uncontrolled tumor growth and metastasis. Overexpression of CXCL8 in the tumor (microenvironment), by infiltrating immune cells, stromal cells, and the tumor cells themselves, promotes the migration of endothelial cells and formation of new blood vessels in the tumor. This neovascularization is essential to supply nutrients and oxygen to the tumor cells and is probably mainly mediated by interaction of CXCL8 with CXCR2-expressing endothelial cells. In addition, CXCL8 may directly stimulate CXCR1- or CXCR2-expressing tumor (microenvironmental) cells, stimulating their survival, proliferation, and migration further enhancing tumor expansion [57]. CXCL8 is also known for its ability to induce epithelial-to-mesenchymal transition of tumor cells. This facilitates tumor cell invasion, metastasis, and resistance to immune cells [104, 105]. Increased CXCL8 expression has been associated with diverse types of tumors. Moreover, several in vivo models and cell lines show the angiogenic, pro-tumorigenic, and pro-metastatic role of CXCL8 and CXCR1/CXCR2 in solid and hematological malignancies. This has been overviewed by some recent reviews [21, 71, 105, 106].

CXCL8 also plays a key role in recruiting neutrophils to the tumor microenvironment. Tumor-associated neutrophils have a dual potential with either tumor-suppressive or tumor-promoting effects. The anti-tumor N1 neutrophil phenotype is characterized by killing of tumor cells and promoting pro-inflammatory immune responses. The pro-tumor N2 neutrophil phenotype is associated with stimulation of angiogenesis, extracellular matrix remodeling by release of proteases, metastasis, and evasion of anti-tumor immune responses [107]. Induction of an immunosuppressive environment is a central feature of growing tumors, which is mediated by tumor cell upregulation of inhibitory immune checkpoint molecules like programmed death-ligand 1 (PD-L1) facilitating immune escape. This is further enhanced by infiltration of CXCR1- or CXCR2-expressing MDSCs, which further promote tumor growth by inhibiting local anti-tumor immune responses mediated by CD8^+^ T-lymphocyte infiltration and cytotoxicity. Tumor-produced CXCL8 can attract both granulocytic as monocytic MDSCs to the tumor microenvironment as shown by in vitro and in vivo models. Moreover, CXCL8 induced the release of NETs in granulocytic MDSCs in vitro [108, 109]. A recent study demonstrated NETs can wrap and coat tumor cells and shield them from immune cytotoxicity by preventing contact with CD8^+^ T-lymphocytes and natural killer cells [110]. Furthermore, CXCL8 can recruit CXCR2-expressing tumor-associated macrophages and induce an immunosuppressive M2 phenotype into the tumor microenvironment, as demonstrated e.g. in a murine model for pancreatic cancer [111]. Additionally, CXCL8 can be involved in the proliferation, self-renewal, and recruitment of CXCR1/CXCR2-expressing cancer stem cells, which are known to promote tumorigenesis, tumor maintenance, metastasis, and drug resistance in many different cancers [71, 105]. Consequently, both systemic and tumor-associated CXCL8 is associated with resistance to or reduced clinical benefit of chemotherapy, radiotherapy, molecularly targeted therapy, or immune checkpoint inhibition therapy like blockade of PD-1 or PD-L1 [112, 113]. Therefore, emerging research suggests that combination therapies of monoclonal anti-CXCL8 antibodies or CXCR1/CXCR2 antagonists with standard anti-tumor (immuno)therapy can tackle the tumor immune escape and provide further benefit in anti-tumor treatment. Efficiency of this approach was demonstrated using in vivo models with some recent examples for breast, lung, prostate, hepatocellular, and pancreatic cancer [114–117]. Moreover, this approach is being investigated in clinical trials for several types of cancer (*vide infra*). Finally, although most of the studies point out a pro-tumorigenic role for CXCL8, there are some reports describing a beneficial role of CXCL8 in the anti-tumor (immune) response in similar cancers, probably mainly induced through leukocyte recruitment [118, 119]. Therefore, it will be required to determine whether the CXCL8-CXCR1/CXCR2 axis will be a good target in personalized anti-tumor treatment.

#### Cardiovascular diseases

Atherosclerosis is a chronic inflammatory disease in which plaque (composed of fats, cholesterol, fibrin, and cellular infiltrates) builds up on the artery walls, which results in narrowing of the blood vessels and blocking of the blood flow. Within atherosclerotic lesions, CXCL8 can be produced by macrophages induced by 25-hydroxycholesterol or coagulation factors. An essential role is played by these monocyte-derived macrophages that form foam cells and oxidize lipoproteins. This increases CXCR2 expression on their surface and makes them responsive for CXCL8-mediated adhesion and chemotaxis. Moreover, the CXCL8-CXCR1/CXCR2 axis and recruited neutrophils contributed to the early phase of atherosclerotic plaque formation in vivo [21]. In contrast, it was shown that CXCR2 activation can direct migration of bone marrow-derived endothelial progenitor cells to regressing atherosclerotic plaques promoting resolution in mice [120]. Besides, CXCR2 can play a role in myocardial ischemia/infarction and arterial hypertension. In murine models, knock-out or blocking of CXCR2 reduced angiogenesis, neutrophil infiltration into, and the size of an infarcted area [121] or prevented experimental hypertension and vascular dysfunction by reducing the recruitment of CXCR2^+^ pro-inflammatory cells [122].

#### Pulmonary diseases

##### Acute lung injury & acute respiratory distress syndrome (ARDS)

Acute lung injury is characterized by direct acute injury to the lungs associated with pulmonary edema and impaired gas exchange, caused by infection, trauma, noxious compounds, systemic inflammation, etc. In acute lung injury, elevated CXCL8 and immune complexes of anti-CXCL8 autoantibodies enhanced neutrophil accumulation and survival, leading to neutrophil-associated damage [123]. CXCR2 antagonists prevented excessive neutrophil recruitment, vascular permeability, lung injury, and impaired gas exchange in several in vivo model systems [21]. Acute lung injury might evolve to ARDS, a severe life-threatening respiratory failure characterized by widespread inflammation and fluid leakage in the lungs. Elevated levels of CXCL8 and neutrophils in the lungs are also observed in ARDS [21]. Using a monoclonal antibody against CXCL8, ARDS-like lung injury and neutrophil infiltration could be diminished in rabbits, making it a valuable target for treatment [124]. Finally, recent attention is paid to the role of CXCL8 and neutrophils in COVID-19 ARDS (*vide infra*).

##### Cystic fibrosis

In sputum, CXCL8 is characterized as an important neutrophil chemoattractant in patients with chronic inflammation of the airways [125]. This includes cystic fibrosis, a progressive autosomal recessive genetic disorder characterized by frequent infections and a declining lung function due to chronic airway obstruction. Neutrophil expression of CXCR1, but interestingly not CXCR2, proved to be important for clearance of bacterial infections in vitro, and removal of CXCR1 from patient neutrophils in the airways due to proteolytic cleavage (mainly by elastase) also impaired bacterial killing. Moreover, glycosylated proteolytic fragments of CXCR1 stimulated bronchial endothelial cells via Toll-like receptor (TLR)2 to produce additional CXCL8. Inhalation of the protease inhibitor alpha1-antitrypsin restored CXCR1 expression and improved bacterial killing in cystic fibrosis patients [21, 126]. Besides, cystic fibrosis patients suffer from airway hyperresponsiveness, in which CXCL8 may induce increased contraction of airway smooth muscle cells [21].

##### Asthma

Asthma is a chronic inflammatory allergic respiratory disease characterized by reversible narrowing of the airways and limited respiratory capacity. Elevated CXCL8, neutrophil, and eosinophil levels are seen in the sputum and bronchial mucosa of asthma patients. Similar to cystic fibrosis, CXCL8 is associated with induction of bronchoconstriction by stimulation of CXCR1/CXCR2-expressing airway smooth muscle cells, as shown in guinea pigs. Besides, CXCR2 may contribute to angiogenesis of peribronchial blood vessels during remodeling of allergic airways through its involvement in migration of bone marrow-derived endothelial progenitor cells. Therefore, blocking CXCL8-CXCR1/CXCR2 interactions could be an interesting therapeutic target for treatment of asthma patients, with evidence in pre-clinical studies [21] and several phase I/phase II clinical trials completed, however with only partial success (*vide infra*).

##### Chronic obstructive pulmonary disease (COPD)

COPD is a chronic inflammatory lung disease, characterized by progressive irreversible airflow obstruction due to fibrosis (chronic bronchitis) or destruction of alveoli (emphysema) and is predominantly caused by smoking. In sputum of COPD patients, elevated CXCL8 levels (mainly secreted by alveolar macrophages) are detected associated with neutrophil (and CXCR2-expressing macrophage) chemotaxis [125, 127]. Excessive release of neutrophil serine proteases and MMPs are directly responsible for the tissue destruction of the small airways and the continued immune cell infiltration [128]. In pre-clinical models of cigarette smoke-induced acute neutrophilic inflammation in the lungs, treatment with CXCR2 antagonists reduced neutrophil infiltration and tissue damage in the airways [129, 130]. Accordingly, several phase I/phase II clinical trials with CXCR1/CXCR2 antagonists or monoclonal anti-CXCL8 antibodies have been completed for treatment of COPD, however with conflicting results so far (*vide infra*).

##### Idiopathic pulmonary fibrosis (IPF)

Finally, increased CXCL8 levels in the airways are also detected in IPF [131], a disease characterized by chronic alveolar fibrosis (excessive collagen deposition) and a decline of the lung function due to abnormal healing of lung injury. Alveolar macrophages might be an important source of the CXCL8 production [132] together with endothelial progenitor cells. Indeed, CXCL8 release by senescent endothelial progenitor cells isolated from IPF patients might contribute to the neutrophil infiltration in the disease [133]. Moreover, CXCL8 and CXCR2 may play an essential role in the pathogenesis of IPF due to their involvement in stimulating angiogenesis and promoting collagen deposition and fibrosis in vivo [134, 135].

#### Transplantation & ischemia-reperfusion injury (IRI)

CXCL8, CXCR1/CXCR2, and neutrophils are associated with rejection of transplanted organs. In type 1 diabetes patients with transplanted pancreatic islets, elevated circulating CXCL8 levels are detected, and CXCR1/CXCR2 activation has been associated with mediating damage to and reducing survival of these islets [136]. In lung transplantation patients, elevated CXCL8 levels and neutrophil counts are detected in broncho-alveolar lavage (BAL) fluids from patients with bronchiolitis obliterans syndrome (BOS) and restrictive allograft syndrome (RAS), two types of chronic rejection and major cause of long-term mortality after lung transplantation [137]. Solid organ transplantation is by definition accompanied by an ischemic period, which is followed by reperfusion. Ischemia already causes local tissue and microvasculature damage whereas restoration of the blood flow is associated with aggravation of the inflammation and consequent excessive damage to the transplanted organ which can become systemic. CXCL8 production is upregulated both during hypoxia and during reperfusion by tissue cells, endothelial cells, and leukocytes which may correlate with the ischemic period of the transplanted organ [21]. CXCL8 leads to infiltration and activation of neutrophils, aggravating the inflammatory response by release of ROS and destructive enzymes. Consequently, downregulation of CXCL8 expression or CXCR2 antagonists reduced neutrophil infiltration and local and systemic inflammation in vivo in the context of kidney [138], liver [139] and lung transplantation [140].

#### Arthritic diseases & pain

Arthritic diseases are rheumatological diseases characterized by (cellular) inflammation and articular damage of the joints. Elevated levels of CXCL8 are seen in synovial fluid and serum of patients with arthritis or gout, associated with increased neutrophil infiltration and hyperactivation in the joints [141–144]. Interestingly, neuropeptides demonstrated to foster CXCL8 production by fibroblast-like synoviocytes and may as such contribute to neutrophilic inflammation and joint damage in rheumatoid arthritis (RA) [145]. In contrast, dopamine could elicit anti-inflammatory effects, as synovial fibroblasts from RA patients showed increased expression of dopamine receptors compared to osteoarthritis patients, with exogenous dopamine stimulation reducing expression levels of CXCL8 in vitro [146]. Besides inducing neutrophil infiltration, CXCL8 probably contributes to the angiogenic activity in the inflamed RA joint, which is vital for efficient leukocyte infiltration and the growth of the RA pannus. Homogenates of human RA synovial tissue, with a significant contribution of CXCL8, elevated endothelial cell chemotaxis and angiogenesis in the rat cornea compared to healthy synovial tissue homogenates [147]. Furthermore, CXCL8 may be involved in regulating pain in the inflamed joints, as blockade of CXCR1/CXCR2 inhibited hypernociception and neutrophil recruitment in a murine model of antigen-induced arthritis [148, 149]. CXCL8 and its association with sympathetic pain was also demonstrated in rats, where it provoked hyperalgesia by a prostaglandin-independent mechanism [150]. Moreover, inhibition of CXCR1/CXCR2 reduced both inflammatory as neuropathic pain (*vide infra)* [151, 152]. Finally, in contrast to most types of arthritis, a homeostatic function for CXCR1/CXCR2 signaling in articular cartilage is suggested to prevent development of severe osteoarthritis. Indeed, disruption of this signaling contributed to the characteristic loss of chondrocyte phenotypic stability [153].

#### Neurological diseases

In the blood of patients with the chronic inflammatory demyelinating disease multiple sclerosis (MS), increased levels of CXCL8, neutrophils, and neutrophil-derived enzymes are detected [154]. In cerebrospinal fluid, higher levels of CXCL8 were observed and correlated to disease activity [155]. By blocking or genetic silencing of CXCR2, neutrophil infiltration in the brain, blood-brain-barrier breakdown, and development of autoimmune demyelination was reduced in experimental autoimmune encephalomyelitis (EAE), a murine model for MS [156]. In contrast, since CXCR2 is also expressed on oligodendrocytes (which are cells responsible for myelin production), some reports indicate that CXCL8-mediated CXCR2 activation might be involved also in the remyelination process. Its putative dual role would make it a challenging target for therapy. However, so far, no consensus is reached with some studies showing no effect at all on remyelination [21, 154, 157]. For Alzheimer’s disease, both beneficial as detrimental functions of the CXCL8-CXCR1/CXCR2 axis have been demonstrated. Elevated levels of CXCL8 were observed in brain tissue lysates protecting human neurons from amyloid-β-induced neurotoxicity in vitro [158]. In contrast, pharmacological inhibition of CXCR2, which is mainly expressed in the microglia in an in vivo model for the disease, impeded microgliosis and oxidative stress and as such led to neuroprotective effects [159].

#### Kidney diseases & diabetes

Increased CXCL8 or CXCR1/CXCR2 expression on infiltrating neutrophils, endothelial cells, and arterial smooth muscle cells correlated with kidney injury and inflammation in glomerulonephritis, nephropathy, nephritis, nephrotic syndrome, and renal cancer. In general, severe neutrophil infiltration and consequent kidney damage could be reduced in vivo by monoclonal antibodies against CXCL8, knock-out of *Cxcr2* or CXCR2 antagonists, providing evidence for potential clinical application [21]. In diabetic kidney disease due to type 2 diabetes, elevated CXCL8 concentrations were detected in patients’ urine and glomeruli tissue, where it might play a role in mediating damage to podocytes, as has been demonstrated in diabetic mice [160]. In another murine model, a CXCR1/CXCR2 antagonist prevented and reversed also type 1 diabetes [161], for which clinical trials are currently ongoing (*vide infra*).

#### Auto-immune diseases

Psoriasis is an auto-immune inflammatory disease causing red, itchy scaly patches on the skin. These skin lesions are characterized by hyperproliferation of CXCR2-expressing keratinocytes and their autocrine stimulation by CXCL8 to release pro-inflammatory molecules (TNF-α, IL-17A, IL-22, IL-33, …). These cytokines are stimulating CXCL8 production leading to infiltration of neutrophils that will also produce CXCL8. CXCL8 and its receptor expression levels as such contribute to disease severity. Moreover, CXCL8 stimulates angiogenesis enhancing cellular infiltration in the skin [21]. High levels of CXCL8 are also found in plasma of patients with SLE correlating with disease activity [162] and in cerebrospinal fluid of neuropsychiatric SLE patients. The impact of CXCL8 in the pathogenesis of SLE has been recently reviewed by Ghafouri-Fard et al. [163]. Moreover, a specific CXCL8 SNP in the promoter region of the *CXCL8* gene has been associated with severe SLE nephritis in African Americans (*vide supra*) [80].

#### Inflammatory bowel diseases (IBD)

Chronic inflammation of the gastrointestinal tract is a hallmark of ulcerative colitis and Crohn’s disease, two types of IBD. In these diseases, CXCL8 is produced by inflamed epithelial cells and both macrophages and neutrophils engage in the inflammation, the latter by producing degrading enzymes and ROS. Inhibition of CXCR2 through knock-out or antagonism exerted beneficial anti-inflammatory effects in vivo but also led to reduced clearance of microbial infections [21]. Moreover, the CXCL8-CXCR1/CXCR2 axis specifically participates in the pathogenesis of ulcerative colitis through multiple signaling pathways, including PI3K/Akt, MAPKs, and NF-κB signaling pathways, as recently reviewed by Zhu et al. [164]. In addition, in ulcerative colitis, a collaboration between the innate and adaptive immune system was recently proposed, as mucosal CD14^+^ monocyte-like cells induced CXCL8 in colonic memory CD4^+^ T-lymphocytes [165].

#### Infectious diseases

Last but not least, neutrophils play an essential role in the clearance of pathogens by phagocytosis, ROS production, and release of antimicrobial proteins, NETs, and enzymes. CXCL8-CXCR1/CXCR2 interaction is one of the main regulators during infection by exerting neutrophil chemotaxis and activation. However, neutrophil activation is also associated with additional collateral damage to healthy tissue, amplifying inflammation and leading to organ dysfunction when not properly resolved. Therefore, therapeutic reduction of excessive neutrophil recruitment to reduce collateral damage in acute and chronic inflammatory diseases always increases the risk for additional burden and reduced clearance of infection, which needs to be monitored and adequately managed.

Special attention is paid to the role of CXCL8 in coronavirus disease 2019 (COVID-19) over the last 3 years. We and others found elevated levels of CXCL8, neutrophils, and neutrophil degranulation products in the blood of hospitalized COVID-19 patients compared to healthy controls [166]. Indeed, an elevated neutrophil-to-lymphocyte ratio has been established as a hallmark of severe COVID-19 [167]. Within BAL fluids, highly increased levels of CXCL8 and other neutrophil attractants were detected in COVID-19 patients in the intensive care unit (ICU) compared to influenza ICU patients. This was associated with elevated hyperactivated neutrophils and severe proteolytic activity in COVID-19 patient lungs [168]. A self-sustaining positive feedback loop of neutrophil-intrinsic and systemic production of CXCL8 may generate activated and pro-thrombotic neutrophils, characterized by degranulation and formation of NETs [169]. Recently, it was demonstrated that anti-chemokine antibodies are associated with a positive outcome in COVID-19 and are predictive for a lack of long COVID symptoms. As such, these antibodies may play an anti-inflammatory role by reducing the damaging inflammatory response associated with neutrophil activation and severe COVID-19 [170].

CXCL8, released by endothelial cells, and CXCR2 also play a key role in neutrophil attraction in sepsis, a potentially life-threatening condition characterized by an extreme response of the body to infection resulting in injury to its own tissues. Due to massive chemokine release, CXCR2 desensitization often impairs adequate control of microbial dissemination leading to systemic inflammation [21]. In addition, neutrophils released increased NETs for microbial clearance in an in vivo sepsis model and after incubation of healthy donor neutrophils with plasma from sepsis patients. Unfortunately, NETs also induce thrombosis and organ injury. Interestingly however, inhibition of CXCR1/CXCR2 signaling reduced NET formation, tissue injury, and mortality but retained bacterial clearance capacity in septic mice [171]. Mechanistically, an essential role for phospholipase D2 (PLD2) in inducing downregulation of CXCR2 expression and inhibition of NET release has been suggested to drive mortality in vivo [172].

### CXCL8 targeted therapy

#### Strategies

Based on in vitro research on patient samples and human cells and in vivo experiments using (gene-deficient) mice, chemokines and their receptors demonstrated to be a promising therapeutic target in many diseases. Most of the clinical research has focused on inhibition of the interaction between chemokines and their receptors using small molecules or neutralizing antibodies targeting the receptors or the chemokines themselves. However, this approach only resulted in limited success, with only three compounds targeting chemokine receptors on the market so far. These are, firstly, the small molecule CCR5 antagonist Maraviroc, which inhibits HIV-1 infection [173]. Secondly, the small molecule CXCR4 antagonist AMD3100 (Plerixafor), which is used as a stem cell mobilizer in patients with non-Hodgkin lymphoma and multiple myeloma [174] and finally mogamulizumab, a humanized antibody against CCR4, which is indicated for the treatment of relapsed or refractory CCR4^+^ adult T‐cell leukemia/lymphoma [175]. Inhibition of the inflammatory effects of CXCL8 could be beneficial for several disorders, both neutrophilic inflammatory as other diseases, since its receptors CXCR1 and CXCR2 are expressed on other cell types as well (*vide supra*). Three strategies can be followed: inhibition of CXCL8 expression, CXCL8-GAG interaction, or CXCL8-receptor interaction. Targeting the interactions between chemokines and GPCRs is not only the most extensively studied but also exploited by viruses and ticks to facilitate their evasion of the immune system [176].

##### Inhibition of CXCL8 expression

Targeting CXCL8 expression could be achieved by inhibiting kinases in signal transduction pathways or transcription factors mediating *CXCL8* gene expression. Several of these inhibitors (e.g. p38 MAPK, MEK, PI3K, JNK, NF-κB, and proteasome inhibitors) were shown to reduce CXCL8 production in leukocytes and different cell lines in vitro. However, in vivo or clinical studies with these compounds have not yet been performed [71].

##### Inhibition of CXCL8-GAG interaction

The CXCL8 (chemokine)-GAG interaction could be an interesting therapeutic target since chemokine binding to GAGs is essential for their in vivo chemotactic activity. Peptides without chemokine receptor signaling properties but with high affinity for GAGs were derived from the chemokines CCL5/regulated on activation, normal T cell expressed and secreted (RANTES), CXCL8, CXCL9/monokine induced by interferon-γ (MIG), or CXCL12γ. These peptides reduced leukocyte (neutrophil) transendothelial migration in response to chemokines like CXCL8 by competing with chemokines for GAG binding. Consequently, they exerted anti-inflammatory effects in several murine acute inflammation models (e.g. gout, antigen-induced arthritis, contact hypersensitivity, …), as recently reviewed by Crijns et al. [4]. In addition, mutated chemokines (CellJammer technology platform) with increased affinity for GAGs (dominant mutations) but impaired capacity to induce receptor signaling (negative mutations), were developed to interfere with GAG-chemokine interactions. A dominant-negative mutated CXCL8 chemokine PA401 displaced wild-type chemokines from GAGs and had anti-inflammatory effects in murine lung inflammation, arthritis, acute renal damage, transplantation, and other animal models of neutrophil-driven inflammation [4, 177]. PA401 was also evaluated in a phase I first-in-human clinical trial (NCT01627002) but was terminated early.

##### Inhibition of CXCL8-receptor interaction

Inhibition of the interaction of CXCL8 with its receptors can be achieved by monoclonal anti-CXCL8 antibodies or CXCR1/CXCR2 antagonists. Their efficiency in preventing neutrophil recruitment and associated inflammatory injury has been proven in several animal models. In combination with the knowhow and broad clinical application of drugs targeting GPCRs, several pharmaceutical companies have developed potent inhibitors over the past decades. However, the translation to humans has unfortunately not been very successful so far. The only compound on the market is a topical formulation of an anti-CXCL8 monoclonal antibody (ABCream, a product of Anogen), used for treatment of the inflammatory skin diseases psoriasis and eczema. This antibody is however only approved in China. The reasons for failure of most clinical trials so far are probably (a) the lack of a complete understanding of the spatiotemporal control of human chemokine biology and activity leading to inappropriate target selection, (b) ineffective dosing, (c) antibodies failing to recognize GAG-bound CXCL8, and (d) difficulties in the transfer of findings in mice to men [178].

#### Pre-clinical models

These difficulties are not difficult to explain. Importantly, mice do not have the gene for the chemokine CXCL8. Instead, they express mouse GCP-2/LIX, which is the murine homolog of human CXCL6, as the most potent neutrophil-activating chemokine. Since murine GCP-2/LIX also signals through both CXCR1 and CXCR2, it is probably the functional murine homolog of human CXCL8. In analogy with human CXCL8, NH_2_-terminal truncation of GCP-2/LIX generates proteoforms that induce more potent neutrophil degranulation and migration in vitro [179]. Furthermore, both natural NH_2_-terminal and COOH-terminal truncations increase the potency to chemoattract murine and human neutrophils in vitro and in vivo. In total, 28 natural murine GCP-2/LIX proteoforms containing 69–92 residues were identified from murine stimulated fibroblasts [180]. Rapid cleavage by mouse gelatinase B (MMP-9) at position Ser4-Val5 potentiated the activity to induce Ca^2+^ mobilization in human neutrophils 2-fold [181]. Interestingly, MMP-8 cleavage of GCP-2/LIX at the same position and at Lys79-Arg80 also increased its biological (chemotactic) activity and was essential for induction of neutrophil recruitment to LPS. As such, it seems that MMP-8 secreted by murine neutrophils initiates a positive feedback loop activating GCP-2/LIX and inducing attraction of new neutrophils promoting LPS responsiveness in murine tissues [182]. This seems similar to the human activation of CXCL8 by neutrophil-derived MMP-9 [181, 183]. Murine homologs for human CXCR1 and CXCR2 have been described [103, 184, 185], but the role and function of murine CXCR1 is hardly understood. Murine CXCR2 is activated by multiple CXC chemokines, whereas the murine CXCR1 homolog is activated by human and murine CXCL6/GCP-2 and CXCL8 [103]. Although CXCL8 is not existing in mice, human CXCL8 still chemoattracts murine neutrophils in vivo [59, 103].

#### Clinical trials

Despite these differences between mice and men, emerging research on chemokine and chemokine receptor biology, together with pharmacokinetic studies used to optimize effective half-life of compounds [186], bring inhibition of CXCL8-receptor interaction one step closer to the clinic. Multiple CXCR1/CXCR2 antagonists or anti-CXCL8 monoclonal antibodies are currently under various stages of clinical development for several inflammatory diseases, which is summarized below. A detailed overview of pre-clinical efficacy and the completed and ongoing clinical trials investigating these compounds is provided in Table 3.

**Table 3:** Overview of compounds inhibiting the CXCL8-CXCR1/CXCR2 interaction in vivo and in completed or ongoing clinical trials

Agent name (company)	Mechanism of action	Targeting diseases	In vivo model/Clinical trials	Results	Ref.
Reparixin/ Repertaxin (Dompé)	CXCR1/2 antagonist	Pancreatic islet transplantation for type 1 diabetes	Diabetic mice	Reduced leukocyte recruitment & improved pancreatic islet engraftment	[136]
			NCT01220856 (Phase II: completed)	Improved transplant outcome	[136]
			NCT01817959 (Phase III: completed)	No generally improved insulin secretion but upon transplantation of sufficient islet mass & use of anti-thymocyte globulin for immunosuppression induction	[301, 302]
		Pancreatic islet auto-transplantation after pancreatectomy	NCT01967888 (Phase II/III: completed)	No improved diabetes outcomes: CXCL8 inhibition alone may be insufficient to prevent damage to transplanted islets	[303]
		Delayed graft function after kidney transplantation	Rat kidney transplantation model	Reduced neutrophil infiltration & prevention of kidney graft function impairment	[138]
			NCT00248040 (Phase II: completed)		
		Early allograft dysfunction after liver transplantation	Murine liver ischemia-reperfusion injury model	Reduced neutrophil recruitment, activation & migration in the liver	[139]
			NCT03031470 (Phase II: completed)		
		Primary graft dysfunction after lung transplantation	NCT00224406 (Phase II: completed)		
		Breast cancer	Human breast cancer xenografts	Reparixin with paclitaxel inhibited tumor growth & metastasis by targeting cancer stem cells	[304, 305]
			NCT02001974 (Phase I: completed)	Reparixin with paclitaxel was safe & partially effective in patients with metastatic breast cancer	[306]
			NCT02370238 (Phase II: completed)	Reparixin with paclitaxel did not prolong progression-free survival	[307]
			NCT05212701 (Phase II: recruiting)		
		COVID-19	Murine models of acute lung injury	Reduced neutrophil infiltration, vascular permeability & tissue injury after LPS stimulation	[308]
			NCT04794803 (Phase II: completed)	Improved clinical outcomes	[187]
			NCT04878055 (Phase III: completed)		
			NCT05254990 (Phase III: recruiting)		
DF2755A (Dompé)	CXCR1/2 antagonist	Pain	Murine models of inflammatory & post-operative pain	DF2755A exerted anti-nociceptive effects	[151]
			Rat model characterized by non-inflammatory peripheral neuropathy	Prevention & reversal of peripheral neuropathic pain by direct inhibition of chemokine-induced excitation of sensory neurons	[152]
		Healthy volunteers	NCT04803396 (Phase I: completed)		
Ladarixin (Dompé)	CXCR1/2 antagonist	Airway inflammation	Murine models of airway inflammation	Reduced neutrophil recruitment & improvement of neutrophil-dependent airway inflammation	[309]
		Healthy volunteers	NCT04854642 (Phase I: completed)		
		Type 1 diabetes	Diabetic mice	Prevention & reversal of type 1 diabetes	[161]
			NCT02814838 (Phase II: completed)	No appreciable effect of short-term treatment on the preservation of residual β-cell function	[188]
			NCT04899271 (Phase II: ongoing)		
			NCT05368402 (Phase II: recruiting)		
			NCT04628481 (Phase III: recruiting)		
SX-682 (Syntrix)	CXCR1/2 antagonist	Advanced solid tumors	Murine models of breast & lung cancer	SX-682 with anti-PD-L1 immunotherapy and TGF-β inhibition reduced EMT of tumor cells & infiltration of granulocytic MDSCs	[114]
			NCT04574583 (Phase I/II: ongoing)		
		Myelodysplastic syndromes	NCT04245397 (Phase I: recruiting)		
		Melanoma	Melanoma xenografts	Reduced tumor growth & MDSC recruitment, enhanced CD8^+^ T-lymphocyte infiltration	[189]
			NCT03161431 (Phase I: recruiting)		
		Colorectal cancer	NCT04599140 (Phase I/II: recruiting)		
		Metastatic pancreatic ductal adenocarcinoma	NCT04477343 (Phase I: recruiting)		
Navarixin/ SCH527123/ MK-7123 (Merck)	CXCR1/2 antagonist	Psoriasis	NCT00684593 (Phase II: completed)		
		Advanced/metastatic solid tumors	Colon cancer xenografts	Inhibition of liver metastasis due to reduced angiogenesis & enhanced apoptosis	[310]
				Reduced tumor growth & improved sensitivity to chemotherapy	[311]
			Melanoma xenografts	Reduced tumor growth & angiogenesis	[312]
			NCT03473925 (Phase II: completed)		
		Asthma	Animal models of pulmonary inflammation	Reduced neutrophil recruitment, mucus production & goblet cell hyperplasia	[313]
			NCT00632502 (Phase II: completed)	Reduced sputum neutrophils	[190]
			NCT00688467 (Phase II: completed)		
Elubrixin/ SB656933 (GSK)	CXCR2 antagonist	Healthy volunteers	NCT00551811 (Phase I: completed)	Elubrixin is safe, well-tolerated & inhibited ozone-induced airway neutrophilia	[314]
			NCT00504439 (Phase I: completed)		
			NCT00615576 (Phase I: completed)		
		Cystic fibrosis	NCT00605761 (Phase I: completed)		
			NCT00903201 (Phase II: completed)	Trends for improvement in sputum inflammatory biomarkers, but no changes in lung function	[191]
Danirixin/ GSK1325756 (GSK)	CXCR2 antagonist	Pulmonary diseases	Rat model	Reduced pulmonary neutrophil recruitment after LPS or ozone challenge	[315]
		Healthy volunteers	NCT01209052 (Phase I: completed)	Danirixin is safe & may have benefit in neutrophil-predominant inflammatory diseases	[316]
			NCT01209104 (Phase I: completed)	Danirixin is safe & may have benefit in neutrophil-predominant inflammatory diseases	[316]
			NCT01453478 (Phase I: completed)	Danirixin is safe & no advantages of a bioenhanced over an immediate-release formulation	[317]
			NCT03136380 (Phase I: completed)	Danirixin is safe	[318]
			NCT03457727 (Phase I: completed)	Food has a negative effect on the pharmacokinetics of danirixin	[319]
			NCT02453022 (Phase I: completed)	Superior biopharmaceutical properties of the hydrobromide salt compared to the free base form	[320]
			NCT01267006 (Phase I: completed)		
			NCT02169583 (Phase I: completed)		
		Healthy volunteers & RSV-infected children	NCT02201303 (Phase I: completed)		
		Influenza	NCT02469298 (Phase II: completed)	Well tolerated & no impedance of viral clearance	[194]
		COPD	NCT02130193 (Phase II: completed)	Improved respiratory symptoms & health status of patients with mild-to-moderate COPD	[192]
			NCT03034967 (Phase II: completed)	Absence of a clear efficacy benefit, unfavorable benefit-risk profile	[193]
AZD5069 (AstraZeneca)	CXCR2 antagonist	Healthy volunteers	NCT00953888 (Phase I: completed)	AZD5069 is safe, rapidly absorbed & twice-daily administration is supported	[321]
			NCT01332903 (Phase I: completed)	Rapid absorption & extensive metabolization	[321]
			NCT01051505 (Phase I: completed)	Steady-state plasma concentrations are achieved within 2–3 days	[321]
			NCT01480739 (Phase I: completed)	Moderate inter- and intra-subject variability in total AZD5069 exposure.	[321]
			NCT01989520 (Phase I: completed)	No major influence of drug formulation on total exposure of AZD5069	[321]
			NCT01100047 (Phase I: completed)	No major influence of ethnicity on total exposure of AZD5069	[321]
			NCT01083238 (Phase I: completed)	No major influence of food or age on total exposure of AZD5069	[321]
			NCT01735240 (Phase I: completed)	Increased total exposure & peak plasma levels upon co-administration of ketoconazole	[321]
			NCT01962935 (Phase I: completed)		
		Asthma	NCT01890148 (Phase I: completed)	AZD5069 is safe & reduced neutrophil recruitment	[322]
			NCT01704495 (Phase II: completed)	No reduced frequency of severe asthma exacerbations	[323]
		Bronchiectasis	NCT01255592 (Phase II: completed)	Reduced sputum neutrophil counts but no improvement in clinical outcomes	[324]
		COPD	NCT01233232 (Phase II: completed)	AZD5069 was well tolerated with no increase in infection rates, supporting further studies	[195]
		Metastatic pancreatic ductal carcinoma	Pancreatic ductal carcinoma murine model	AZD5069 (and immunotherapy) extended survival & reduced metastasis	[196]
			NCT02583477 (Phase I/II: completed)		
		Metastatic castration-resistant prostate cancer	Prostate cancer xenograft	AZD5069 in combination with radiotherapy attenuated tumor growth & progression	[115]
			NCT03177187 (Phase I/II: ongoing)		
		Advanced solid tumors & head and neck carcinoma	NCT02499328 (Phase I/II: ongoing)		
AZD4721/ RIST4721 (AstraZeneca)	CXCR2 antagonist	Healthy volunteers	NCT01889160 (Phase I: completed)		
			NCT04105959 (Phase I: completed)		
			NCT05023811 (Phase I: completed)		
			NCT01962935 (Phase I: completed)		
		Palmoplantar pustulosis	NCT03988335 (Phase II: completed)	Well tolerated supporting further clinical development	[197]
			NCT05194839 (Phase II: recruiting)		
		Hidradenitis suppurativa	NCT05348681 (Phase II: recruiting)		
		Familial Mediterranean Fever	NCT05448391 (Phase II: recruiting)		
ABX-IL8 (Abgenix)	Anti-CXCL8 monoclonal antibody	COPD/chronic bronchitis	NCT00035828 (Phase II: completed)	No clinical impact on COPD exacerbations, lung function & health-related quality of life	[198]
		Melanoma	Melanoma xenograft	Inhibition of angiogenesis, tumor growth & metastasis	[199]
		Bladder cancer	Orthotopic bladder cancer xenografts	Reduced tumor growth through downregulation of MMPs & NF-κB	[200]
HuMax-IL8/ BMS-986253 (Bristol-Myers Squibb)	Anti-CXCL8 monoclonal antibody	Advanced solid tumors	Breast cancer xenografts	Reduced tumor infiltration of granulocytic MDSCs & reversal of EMT	[201]
			NCT02536469 (Phase I: completed)	HuMax-IL8 is safe & well tolerated	[325]
			NCT04572451 (Phase I: recruiting)		
			NCT03400332 (Phase I/II: recruiting)		
		Pancreatic carcinoma	Humanized pancreatic cancer murine model	Potentiation of the anti-tumor effects of immunotherapy	[117]
			NCT02451982 (Phase II: recruiting)		
		Hepatocellular carcinoma	NCT04050462 (Phase II: ongoing)		
		NSCLC & hepatocellular carcinoma	NCT04123379 (Phase II: recruiting)		
		Head & neck squamous cell carcinoma	NCT04848116 (Phase II: recruiting)		
		Prostate cancer	NCT03689699 (Phase I/II: ongoing)		
		Myelodysplastic syndromes	NCT05148234 (Phase I/II: recruiting)		

This table provides an overview of compounds evaluated in pre-clinical research, completed clinical trials, and trials that are currently ongoing. When a clinical trial is completed and no results are described, then no published results were found

*COPD* chronic obstructive pulmonary disease, *EMT* epithelial-to-mesenchymal transition, *GSK* GlaxoSmithKline, *MDSC* myeloid-derived suppressor cell, *MMP* matrix metalloproteinase, *NF-κB* nuclear factor kappa-light-chain-enhancer of activated B cells, *NSCLC* non-small cell lung cancer, *PD-L1* programmed death-ligand 1, *RSV* respiratory syncytial virus

##### CXCR1/CXCR2 antagonists

Dompé Farmaceutici developed three different small molecule, non-competitive allosteric antagonists of both CXCR1 and CXCR2. Reparixin, also known as repertaxin, was the first compound used in phase I to phase III clinical trials for transplantation, cancer, and COVID-19 treatment. For transplantation and cancer, only limited results are published but inconclusive, unconfirmed, or no major beneficial effects were found so far. In patients with severe COVID-19 treated with plerixafor, an improvement in clinical outcomes was observed compared to standard of care [187]. Larger phase III studies are currently ongoing to confirm these results. DF2755A is a second CXCR1/CXCR2 antagonist developed by Dompé and is only recently being investigated in a clinical trial after successful in vivo application for treatment of pain [151, 152]. Finally, ladarixin is evaluated in novel phase II/III studies in which the efficacy of this CXCR1/CXCR2 antagonist in longer-term therapy of type 1 diabetes will be assessed, after no effect was seen with short-term treatment only (Table 3) [188].

Syntrix Pharmaceuticals is evaluating SX-682; an oral, small-molecule, allosteric inhibitor of CXCR1 and CXCR2. With this compound, five phase I/phase II clinical trials are currently ongoing or recruiting participants for investigation of safety and efficacy (often in combination with immunotherapy) in cancer treatment after successful murine experiments (Table 3) [114, 189].

Navarixin, also known as SCH527123 or MK-7123, is an allosteric, non-competitive and orally active antagonist of both CXCR1 and CXCR2 developed by Merck. This compound was evaluated in four phase II trials for treatment of psoriasis, cancer, and asthma. For the latter, promising in vivo and clinical trial results were published but larger studies of longer duration are desired (Table 3) [190].

##### CXCR2 antagonists

Elubrixin (also known as SB656933) and danirixin (also known as GSK1325756) are two competitive, reversible and orally active small molecule selective CXCR2 antagonists developed by GlaxoSmithKline. For elubrixin, phase I studies and a phase II trial for treatment of cystic fibrosis have been completed. The drug was well tolerated but requires further studies for adequate efficacy evaluation [191]. Danirixin is evaluated in the context of viral diseases and COPD. After confirming its safety, efficacy studies for use in COPD treatment were conflicting and lack a clear benefit in influenza patients so far (Table 3) [192–194].

AstraZeneca also developed two oral, selective, and reversible CXCR2 antagonists. For AZD5069, phase II clinical trials for treatment of pulmonary diseases (COPD, asthma, and bronchiectasis) and cancer have been initiated after several positively evaluated safety and pharmacokinetic studies. For asthma and bronchiectasis, no clear improvements in clinical outcome were observed but a COPD trial supports further studies [195]. For cancer, clinical trials are still ongoing or no results are released yet, but in vivo results look promising [115, 196]. The second CXCR2 antagonist, AZD4721 (also known as RIST4721), is evaluated in phase II studies for two types of chronic inflammatory skin diseases and for Familial Mediterranean fever (FMF). For palmoplantar pustulosis, further clinical development is supported (Table 3) [197].

##### CXCL8-neutralizing antibodies

Besides inhibition of the CXCL8 receptors, two antibodies targeting CXCL8 are being investigated in clinical trials. The first one is ABX-IL8, a fully humanized anti-CXCL8 monoclonal antibody from Abgenix. However, this antibody was not successful so far for treatment of COPD, psoriasis, or RA [198]. For melanoma and bladder cancer, promising in vivo results were obtained [199, 200], but no clinical trials have been performed yet for these diseases. The second antibody is HuMax-IL8 (BMS-986253), a fully human anti-CXCL8 monoclonal antibody from Bristol-Myers Squibb. It was found to be safe and well tolerated and cancer patients are currently part of or being recruited for eight other phase I/phase II studies, which will evaluate treatment combinations of immunotherapy or radiotherapy with HuMax-IL8 after successful in vivo studies (Table 3) [117, 201].

### The connection of CXCL8 with the chemokine CXCL12

The inflammatory chemokine CXCL8 has an interesting connection with the traditionally considered homeostatic chemokine CXCL12. Together, they are regulating the process of retention of neutrophils in and release from the bone marrow and can act synergistically in inflammatory conditions.

#### Retention of neutrophils in & release from the bone marrow

Neutrophils undergo differentiation and maturation from hematopoietic stem cells into mature segmented neutrophils in the bone marrow. Activation of CXCR2 signaling on mature neutrophils, mediated by CXCL1 to 3 and CXCL5 to 8, induces release in the peripheral circulation. As such, it directly antagonizes the function of CXCR4, which by interacting with its ligand CXCL12 expressed by bone marrow stromal cells, retains CXCR4-expressing hematopoietic progenitor and stem cells and the developing neutrophils in the bone marrow [202]. CXCR4 expression is typically downregulated by stimulation with the myelopoietic growth factor granulocyte-colony stimulating factor (G-CSF), which promotes the neutrophil mobilization process [203]. Moreover, G-CSF can decrease CXCL12 production and upregulate CXCR2-binding chemokines, all together initiating neutrophil release in the circulation [204, 205]. In contrast, the cytokine IL-4 impairs neutrophil migration, which could counteract the neutrophil release [206]. Interestingly, in mice, G-CSF can also counteract CXCR2-mediated signaling preventing excessive neutrophil release after an initial acute phase of rapid neutrophil mobilization induced by CXCR2 ligands [207]. Finally, upregulation of CXCR4 and downregulation of CXCR2 in aging neutrophils might be important for their return (homing) to the bone marrow for clearance by resident macrophages, which is demonstrated in mice [208, 209].

#### Synergy of CXCL8 with CXCL12 for leukocyte chemotaxis

CXCL12 can dose-dependently enhance neutrophil migration toward a suboptimal concentration of CXCL8 in vitro and to GCP-2 in inflamed peritoneum in vivo [100, 102]. Moreover, CXCL8 together with CXCL12 enhanced chemotaxis of CXCR1-transfected, CXCR4-positive Jurkat cells. As previously mentioned, this synergistic effect could be reduced by the CXCR4 antagonist plerixafor, indicating CXCL12 needs to activate its own receptor to observe the synergistic effect [100]. Besides, the synergy between CXCL8 and CXCL12 was demonstrated in the migration of monocyte-derived immature dendritic cells [210] but not of monocytes themselves [211]. As such, CXCL12 is not only a homeostatic chemokine but also exerts an inflammatory function in specific conditions.

## The homeostatic & inflammatory chemokine CXCL12

**Fig. 6:**
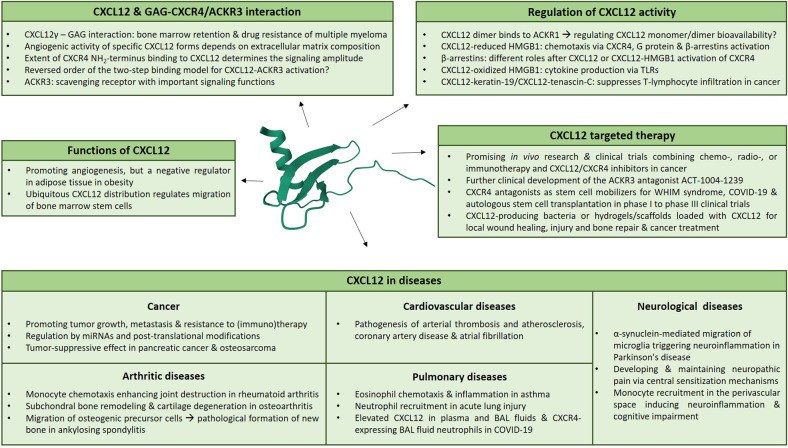
Cutting-edge research progress in the field of CXCL12. The solution NMR structure of the CXCL12 monomer was drawn from PDB accession code 2KEC. BAL fluid broncho-alveolar lavage fluid, GAG glycosaminoglycan, HMGB1 high-mobility group box 1, WHIM syndrome Warts, Hypogammaglobulinemia, Immunodeficiency, and Myelokathexis syndrome

Due to the interesting relationship of CXCL8 with CXCL12, we end this review with a discussion of research in the field of CXCL12 that was performed over the last few years (Fig. 6), after publication of our two review papers in 2018 [9, 10].

### CXCL12 & its receptors CXCR4, ACKR3 & ACKR1

In contrast to *CXCL8*, the human *CXCL12* gene located on chromosome 10q11 codes for six splicing variants. They share the first three exons and have a different 4^th^ exon, generated by alternative splicing. The transcription and translation process of these splicing variants has been located to certain tissues and specific variants are linked to specific biological activities after translation. For instance, CXCL12γ is the form with the most potent anti-HIV-1 activity and the most potent chemoattractant in vivo. It differs from other CXCL12 forms due to its unique extended COOH-terminal domain with mostly positively charged amino acids and binds with the highest affinity to GAGs [9, 10]. Recently, CXCL12γ, and not CXCL12α, was specifically associated with immobilization on heparan sulfate on bone marrow stromal cells, where it controls adhesion/retention of multiple myeloma cells and provides drug resistance by this interaction [212]. Besides, using microfluidic biomimicry integrated with three-dimensional extracellular matrix hydrogels (a way to reconstitute blood vessel-like analogs in vitro), it was demonstrated that angiogenesis exerted by specific CXCL12 forms is determined by the matrix composition. The CXCL12α form (compared to CXCL12β and CXCL12γ) displayed the highest potency in promoting specific angiogenesis (blood vessel sprouting) and vascular permeability in collagen-only hydrogels, whereas CXCL12γ became more potent after addition of the GAG hyaluronic acid [213]. After transcription, translation, and removal of the signal peptide, a protein of 68 amino acids (for CXCL12α) is generated, which can be extended by 1 to 51 residues for other CXCL12 forms [9, 10].

CXCL12 interacts with the seven-transmembrane receptors CXC chemokine receptor 4 (CXCR4) and atypical chemokine receptor 3 (ACKR3), also known as chemokine receptor RDC-1 or CXCR7. Recently, also interaction of CXCL12 with ACKR1 has been demonstrated (*vide infra*). CXCR4 is a GPCR with CXCL12 as the only chemokine ligand and expressed on most leukocytes, hematopoietic progenitor and stem cells, keratinocytes, endothelial cells, epithelial cells, and stromal cells in the bone marrow, thymus, and lymph nodes. As such, CXCR4 is probably the most widely expressed GPCR for chemokines. CXCR4-mediated signaling is mainly G protein-mediated, resulting in activation of the MAPK, PLC, and PI3K pathways, Ca^2+^ mobilization, and eventual activation or migration of the target cell. It was also suggested that CXCR4 can signal independently of G proteins after dimerization, through activation of Janus kinase (JAK)2 and JAK 3 and downstream signal transducer and activator of transcription (STAT) phosphorylation, inducing Ca^2+^ mobilization and chemotaxis. However, other researchers disagreed with these findings [9]. Over the last years however, CXCL12-induced JAK2/STAT3 signaling in human non-small-cell lung cancer cells and JAK2/STAT3 and JAK3/STAT6 signaling in a human proximal tubular cell line was demonstrated. This was specifically associated with resistance to chemotherapy and aggravation of renal fibrosis, respectively [214, 215]. Interestingly, also the enhanced migratory response of monocytes from patients with active RA in response to the CXCL12 - high-mobility group box 1 (HMGB1) heterocomplex (*vide infra*) was dependent on JAK2 signaling [216]. In addition, CXCL12 binding to CXCR4 also induces β-arrestin recruitment, which can sterically block other signaling pathways and initiate receptor and CXCL12 internalization and degradation, but can also directly activate p38 MAPK signaling which further promotes CXCR4-mediated chemotaxis [9].

CXCL12, together with CXCL11, also binds (with ten-fold higher affinity than for CXCR4) to ACKR3, which is expressed on hematopoietic and neuronal progenitor cells and activated endothelial cells. ACKR3 is an atypical chemokine receptor inducing β-arrestin recruitment. The signaling bias for arrestin and failure to couple to G proteins may be caused by a specific pose of ACKR3-bound CXCL12, as recently demonstrated by Yen et al. [217]. ACKR3 mainly acts as a scavenger receptor removing CXCL12 from the environment by continuous internalization and recycling of ACKR3. In contrast to CXCR4, binding to ACKR3 does not lead to receptor degradation, favoring the continuous cycles of ACKR3 between the plasma membrane and vesicles in the cytoplasm [9]. The clearance and lysosomal degradation of CXCL12 via ACKR3 binding and internalization was proposed to be part of a negative-feedback loop, aiming to maintain optimal CXCL12 concentrations. This may be required to achieve continuous robust directional cell migration which was recently confirmed in a zebrafish model [218]. However, β-arrestin recruitment after ACKR3 activation may also potentiate cell migration through cooperation with CXCL12-activated CXCR4, although the precise mechanism is not clear yet [219]. In addition, ACKR3 induced G protein-independent MAPK signaling associated with migration of T-lymphocytes and neural progenitor cells [9] and resistance to prostate and non-small cell lung cancer treatment [220, 221]. Finally, ACKR3 has been characterized as a broad-spectrum scavenger receptor for several families of opioid peptides, regulating their availability for classical opioid receptors and thereby modulating pain [222]. In conclusion, ACKR3 is much more than a simple scavenging receptor as it also has important signaling functions which are far from completely elucidated.

### Interaction of CXCL12 with receptors & GAGs

In the CXCL12 chemokine structure, two CXCR4-binding domains are described in the two-step/two-site binding model. The first domain comprises the first eight NH_2_-terminal amino acids of CXCL12 whereas the second domain RFFESH is located COOH-terminally of the CXC motif and close to the first domain. The RFFESH domain is believed to be responsible for initial binding between CXCL12 and CXCR4, providing binding affinity and inducing a conformational change after which the NH_2_-terminus of CXCL12 activates signaling in the CXCR4 receptor-binding pocket [9]. However, similar to CXCL8 binding to CXCR1/CXCR2, this interaction is probably much more complex than initially anticipated in the two-step/two site model [223]. Indeed, using a systematic mutagenesis strategy, it was recently found that the NH_2_-terminus of CXCR4 not only provides binding affinity but also contributes to the CXCL12 signaling, with the signaling amplitude depending on the extent to which the NH_2_-terminus of CXCR4 binds CXCL12 [224]. Moreover, within CXCL12, Pro10 (the “X” in the CXC motif) was found to be crucial to exert efficient CXCR4 binding and agonist activity whereas deletion of this Pro residue had negligible effect on binding to the CXCR4 NH_2_-terminus only. Probably, the Pro10 residue optimally positions the CXCL12 NH_2_-terminus for efficient docking into the receptor pocket and activation of CXCR4 [225].

For CXCL12 binding to ACKR3, an alternative for the classical two-step binding model of chemokines to receptors was projected. It was found that the NH_2_-terminus of ACKR3 plays a key role in CXCL12 binding and receptor activation by preventing chemokine dissociation. As such, a reversed order was proposed, in which the NH_2_-terminus of CXCL12 initially forms interactions with the extracellular loops and transmembrane pocket of ACKR3, followed by the receptor NH_2_-terminus wrapping around the core of CXCL12, which prolongs its residence time [226]. For the first stage of this interaction, NH_2_-terminal residues 3–5 of CXCL12 and the transmembrane pocket formed by helices 2, 6, and 7 of ACKR3 were determined to be critical [227].

Like for most chemokines, interaction of CXCL12 with GAGs is indispensable for its in vivo activity, protecting it from proteolysis and being essential for immobilization, chemotaxis, angiogenesis, and other functions (*vide infra*). A typical heparin GAG-binding domain is described in the first β-strand of CXCL12, being a cluster of the amino acids Lys24, His25, Leu26, and Lys27 [9]. However, based on three-dimensional structure interactions, also other residues are characterized to be involved in the interaction with GAGs including Arg20, Ala21, Asn30, Arg41, and Lys64 [4]. Probably, also some positively charged amino acids in the NH_2_-terminus are involved since NH_2_-terminal proteolysis reduces the affinity for GAGs (*vide infra*).

### Functions of CXCL12

CXCL12 is traditionally considered as a homeostatic chemokine, as it is involved in physiological processes like embryogenesis, neurogenesis, cardiogenesis, hematopoiesis, leukocyte homing, and angiogenesis, by inducing migration and activation of hematopoietic progenitor and stem cells, endothelial cells, and most leukocytes [9, 10]. Interestingly, a recent study using cultured adipose tissue and obese mice suggested that CXCL12 can also block angiogenic processes. CXCL12-CXCR4/ACKR3 interaction negatively regulated angiogenesis in adipose tissue by inhibiting platelet-derived growth factor-B-induced pericyte dissociation from blood vessels, which normally promotes vascular remodeling for expansion of adipose tissue in obesity [228]. As mentioned before, CXCL12 also retains hematopoietic progenitor and stem cells in the bone marrow. Interestingly, it was shown in situ that no CXCL12 long-range gradients but a ubiquitous CXCL12 distribution with local enrichments controls cellular migration in the bone marrow [229]. Besides its homeostatic function, CXCL12 is also involved in regulation of inflammation, which is illustrated by its role in wound healing and tissue repair and involvement in inflammatory diseases (*vide infra*).

### Regulation of CXCL12 activity

Like CXCL8, also CXCL12 activity is complex and regulated at multiple levels. Transcription, alternative splicing, mRNA stabilization, and translation are tightly controlled processes and influence the CXCL12 activity. For instance, hypoxia upregulates while TGF-β downregulates the expression of CXCL12. After translation, post-translational modifications further influence the activity of CXCL12. In contrast to CXCL8, all post-translational modifications of CXCL12 are known to reduce or abrogate its biological (chemotactic) activity and reduce the affinity for GAGs. Proteolytic removal of NH_2_- or COOH-terminal amino acids (by CD26, dipeptidyl peptidase 8, leukocyte elastase, MMPs, cathepsin G, carboxypeptidase N, carboxypeptidase M, or cathepsin X), citrullination of multiple arginine residues by peptidyl arginine deiminase, or nitration of one or two tyrosine residues are described. In general, COOH-terminal truncation more moderately reduces the activity of CXCL12 compared to NH_2_-terminal processing, but makes CXCL12 more susceptible to these NH_2_-terminal truncations [9, 10].

Once produced, GAG binding and homo- or hetero-dimerization of both CXCL12 and its receptors (even without ligand stimulation) are influencing downstream signaling and functional activity. Also GAG binding itself is promoting the CXCL12 homo- and hetero-dimerization process, which further promotes GAG interactions [9]. Whereas CXCR4 and ACKR3 prefer binding of monomeric forms, the CXCL12 dimer, and not the monomer, was recently shown to bind to the extracellular NH_2_-terminus of ACKR1 with low nanomolar affinity and can be internalized by this receptor on ACKR1-transfected cells. Moreover, primary erythrocytes expressing ACKR1 are efficiently bound by the dimeric form of CXCL12. This suggests that this interaction might be a novel pathway in the regulation of the relative bioavailability of monomeric and dimeric forms of CXCL12 [230].

Heterodimerization of CXCL12 has been characterized with platelet chemokines (CCL5, CXCL4, and CXCL7) and with the alarmin HMGB1, a nuclear protein released by necrotic and severely stressed cells. The interaction of HMGB1 with CXCL12 was demonstrated to be necessary for the attraction of monocytes to tissue injury sites through CXCR4 activation [9, 231]. CXCL12-HMGB1 bound as a balanced agonist to CXCR4, inducing both G protein- and β-arrestin-mediated signaling pathways to sustain chemotaxis. More specifically, using knock-out cells, it was found that primarily β-arrestin 1 regulates actin polymerization after CXCL12-HMGB1 activation of CXCR4, whereas both β-arrestin 1 and 2 do so after CXCL12 activation. Conversely, both β-arrestins are involved in the induction of chemotaxis after CXCL12-HMGB1 activation whereas only β-arrestin 1 regulates migration after CXCL12 activation of CXCR4. Moreover, β-arrestin 2 activation surprisingly retains CXCR4 on the cell surface after CXCL12-HMGB1 activation, while it internalizes CXCR4 for degradation after CXCL12 activation. As such, β-arrestins may play distinct roles dependent on the ligand that triggers the CXCR4 receptor [232]. Moreover, HMGB1 needs to be in its reduced form when interacting with CXCL12 to exert chemotactic activity on monocytes via CXCR4 interaction, as the oxidized HMGB1 form binds to TLR2 and TLR4, leading to production of cytokines and chemokines. The redox potential of the microenvironment could as such be essential for the chemotactic activity of the heterocomplex. Indeed, an active thioredoxin system was required to sustain inflammation in rheumatoid arthritis [216]. In skeletal muscle, the oxidized HMGB1 form is associated with inflammation in muscular dystrophy diseases, which is combined with a loss of tissue regeneration induced by the reduced HMGB1-CXCL12 complex. As such, rebalancing the expression of HMGB1 redox isoforms could be a therapeutic strategy to counteract muscular dystrophy [233]. Targeting the CXCL12-HMGB1 heterocomplex for therapy was also described with diflunisal, an aspirin-like nonsteroidal anti-inflammatory drug, that inhibited the chemotactic activity of HMGB1 at nanomolar concentrations both in vitro and in vivo by disrupting the heterocomplex [234]. Peptide inhibitors targeting the CXCL12/HMGB1 interaction could also have therapeutic potential [235]. Besides, an interesting heterodimerization of CXCL12 with keratin-19 was characterized in pancreatic, colorectal, and breast cancer carcinomas. This heterodimer formed a filamentous coating on the tumor, suppressing T-lymphocyte infiltration [236]. Moreover, tenascin-C, an extracellular matrix component, immobilized infiltrating T-lymphocytes through inducing expression and binding of CXCL12 in vitro [237]. These interactions may explain the failure of cancer immunotherapy and as such promote cancer progression in patients. Finally, CXCL12 has been characterized to form a heterodimer with the lectin galectin-3, which modulated inflammation by attenuating CXCL12-induced CXCR4 signaling and leukocyte chemotaxis [238].

As a final level of activity regulation, CXCL12 synergizes with CXCL8 (*vide supra*) but also with CXCL9, CXCL10, CXCL11, and multiple CC chemokines to attract B- and T-lymphocytes, dendritic cells, monocytes, and CD34^+^ progenitor cells [101].

### CXCL12 in diseases

Differential expression of CXCL12 (splicing variants) or CXCR4/ACKR3 has been demonstrated in various diseases with CXCL12 having either beneficial or harmful effects [10]. Although mice possess fewer splicing variants of CXCL12, human and mouse CXCL12 show an exceptional homology on both genome and protein level. As such, mice are an appropriate model organism for the investigation of CXCL12 in various pathologies. Here, we provide an update on the role of CXCL12 in a few major disease categories.

#### Cancer

Most research on the role of CXCL12 in disease has been focusing on cancer. Indeed, as CXCL12 is upregulated by hypoxia, it is abundantly detected in tumors. CXCL12-CXCR4/ACKR3 interactions promote tumor growth, adhesion, vascularization, invasion, and metastasis to CXCL12-rich organs like lungs, liver, bone marrow, and lymph nodes [10]. These tumor promoting effects were recently confirmed in several types of hematologic malignancies [239] and solid tumors [240]. CXCL12 stimulation also induces resistance to chemo- and radiotherapy by maintaining and stimulating CXCR4-expressing adhesive cancer cells in a protective microenvironment like the bone marrow, as has been recently confirmed for hematological malignancies [239] and pancreatic cancer [241]. Moreover, resistance to immunotherapy, due to reduced tumor infiltration of cytotoxic T-lymphocytes or increased infiltration of MDSCs, was induced by CXCL12 signaling in vitro and in vivo, resulting in further promotion of tumor growth [242, 243]. Although scarce, there are some recent reports associating CXCL12 with tumor-suppressing effects. Using patient samples and an in vivo model, it was demonstrated that osteosarcomas can epigenetically downregulate CXCL12 expression and as such acquire the ability to form metastases and impair cytotoxic T-lymphocyte homing to the tumor site [244]. Moreover, reduced CXCL12 signaling contributed to resistance of pancreatic cancer subpopulations to T-lymphocyte-mediated cytotoxicity [245], contrasting the study of Garg et al. [242].

However, the predominant tumor-promoting effects of CXCL12 are tightly regulated. Regulation of CXCL12 expression by miRNAs can play a significant role in either tumor progression or suppression, dependent on the miRNA type. A recent study suggested a role for exosomal miR-146a-5p and miR-155–5p promoting CXCL12-induced metastasis of ACKR3-expressing colorectal cancer cells. These exosomal miRNAs were internalized and promoted the activation of cancer-associated fibroblasts, which increase the invasive and metastatic capacity of the cancer cells by *i.a*. enhancing levels of CXCL12 [246]. Moreover, exosomal transfer of miRNA from CXCR4-overexpressing colorectal cancer cells to tumor-associated macrophages was shown to enhance their M2 polarization and metastasis-promoting characteristics [247]. In contrast, in a murine model of colitis-associated colorectal cancer, miR-126 (which was downregulated in patients with colorectal cancer) reduced CXCL12 expression in intestinal epithelial cells functioning as a tumor suppressor by inhibiting the recruitment and function of macrophages [248]. In colorectal cancer, it is however clear that the CXCL12-CXCR4/ACKR3 axis itself has tumor- and metastasis-promoting effects, which were recently reviewed by Goïta et al. [249].

Finally, post-translational modifications of CXCL12 can influence cancer progression. As the dipeptidyl peptidase CD26 is associated with inactivation of CXCL12, absent or lower CD26 expression and activity in the tumor can result in a defective truncation and prolonged activity of CXCL12. This may further promote cancer cell growth, angiogenesis, metastasis, and homing to or retention in the bone marrow [250].

#### Cardiovascular diseases

Recent in vitro and in vivo evidence indicated that interaction of platelet-derived CXCL12 with CXCR4 participates in the pathogenesis of arterial thrombosis. Inhibition of CXCR4 attenuated platelet aggregation induced by collagen or human plaque homogenates under static and arterial flow conditions. Mechanistically, CXCR4 signaling activated the Bruton’s tyrosine kinase pathway, leading to integrin activation and platelet aggregation. Moreover, CCL5-derived peptides binding to CXCL12 inhibited Bruton’s tyrosine kinase signaling and the induced platelet aggregation, which could have therapeutic value in atherothrombosis [251]. Using knock-out mice and human coronary artery endothelial cells, endothelial ACKR3 was suggested to contribute to the pathogenesis of atherosclerosis by promoting the adhesion to and invasion of immune cells through arterial vascular endothelium [252]. Additionally, blood CXCL12, together with macrophage colony-stimulating factor 1, were identified as causal mediators of coronary artery disease [253]. Finally, CXCL12 may play a key role in atrial fibrillation since antagonizing CXCR4 with plerixafor in vivo prevented the development and duration of the disease. This was mediated by reducing T-lymphocyte and macrophage infiltration and ERK/AKT signaling in the murine atria [254].

#### Arthritic diseases

CXCL12 sustains and promotes the progression of rheumatoid arthritis. It has been associated with inducing aberrant joint neoangiogenesis, CXCR4^+^ T-lymphocyte and osteoclast chemotaxis and activation, and necrosis of cartilage-protecting chondrocytes, which leads to chronic joint inflammation and destruction [10]. On top of that, a recent study showed hypoxia-mediated upregulation of CXCL12 in murine fibroblast-like synoviocytes enhancing the recruitment of CXCR4-expressing monocytes which increased synovial inflammation and bone erosion [255]. Moreover, fibroblast growth factor receptor 3 (FGFR3) deficiency in murine myeloid cells enhanced CXCL12-dependent chemotaxis of macrophages via upregulation of ACKR3, resulting in increased joint destruction [256]. In osteoarthritis, CXCL12 induced uncontrolled patient-derived osteoblast proliferation and release of cartilage-degrading enzymes by and necrosis of chondrocytes [10]. As such, CXCL12 may be involved in the aberrant subchondral bone remodeling. These effects seem to be partially mediated by modulation of CXCL12-ACKR3 signaling by the proton-activated GPCR GPR4, which is abundantly expressed in human and mice osteoarthritis cartilage and proved to be essential for the development of osteoarthritis [257]. Moreover, activation of mechanistic target of rapamycin complex 1 (mTORC1) in subchondral bone pre-osteoblasts promoted secretion of CXCL12, which induced subchondral bone remodeling and cartilage degeneration [258]. Finally, a recent in vivo study demonstrated that CXCL12-CXCR4 stimulation induced migration of osteogenic precursor cells contributing to pathological formation of new bone in ankylosing spondylitis, a form of chronic arthritis characterized by chronic inflammation of the joints of the spine [259].

#### Pulmonary diseases

CXCL12 is known to aggravate asthma as it is associated with stimulation of eosinophilia, neovascularization, and attraction of progenitor cells and leukocytes in inflamed lungs. Use of CXCR4-neutralizing antibodies or intranasal administration of plerixafor in vivo demonstrated to be successful in reducing these effects [10]. Recently, also an orally active CXCL12 neutraligand known to neutralize the activity of CXCL12, pyrimidinone 57 (LIT-927), reduced eosinophil chemotaxis and exerted anti-inflammatory effects in a murine model of allergic airway hypereosinophilia [260]. In acute lung injury, CXCL12, although not a typical neutrophil attractant, is associated with chemoattraction of CXCR4-expressing neutrophils and suppression of murine neutrophil apoptosis in vitro aggravating the lung injury [10]. A recent study demonstrated that CXCL12 is essential for neutrophil recruitment in the early phase of NLR family pyrin domain containing 3 (NLRP3)-mediated neutrophilic lung injury, with the levels of CXCL12 regulated by the NLRP3 inflammasome [261]. Finally, in COVID-19, CXCL12 plasma levels were shown to be significantly elevated in patients in ICU compared to healthy volunteers [166] and patients with mild COVID-19 [262]. Moreover, blood neutrophils from COVID-19 patients were hyper-responsive to CXCL12 in shape change assays in comparison to neutrophils from healthy controls [166]. In BAL fluids, elevated levels of CXCL12 in ICU patients with severe COVID-19 compared to ICU patients with influenza were observed, together with upregulation of CXCR4 on COVID-19 BAL fluid neutrophils [168].

#### Neurological diseases

In neurodegenerative disorders, CXCL12 has both pro-inflammatory and anti-inflammatory effects. CXCL12 exerted protective anti-inflammatory effects in Alzheimer’s disease (e.g. promoting microglial phagocytosis of amyloid beta plaques) but attracted pro-inflammatory microglia involved in the disease onset and progression of amyotrophic lateral sclerosis (ALS) [10]. Recently, it was shown that CXCL12 is involved in the α-synuclein-mediated migration of microglia and consequent triggering of neuroinflammation in Parkinson’s disease [263]. In MS, CXCL12 presumably plays both anti-inflammatory as protective effects as it is associated with redistribution of perivascular CXCL12 via ACKR3 from the basolateral to the luminal side of the blood brain barrier during disease onset but also with later induction of remyelination and regulatory T cells [10]. Moreover, CXCL12/CXCR4 signaling may develop and maintain neuropathic pain via central sensitization mechanisms [264]. Neuropathic pain has been associated with cognitive impairment, at least partially caused by neuroinflammation. In this context, it was shown that CXCL12 mediates monocyte recruitment into the perivascular space, which is critical for the induction of neuroinflammation and the resultant cognitive impairment [265].

### CXCL12 targeted therapy

Blocking the CXCL12-CXCR4/ACKR3 interaction by administration of small-molecule antagonists or neutralizing antibodies showed to be beneficial in pre-clinical and clinical studies for treatment of cancer, viral infections, IBD, arthritis, asthma, acute lung injury, ALS, and Warts, Hypogammaglobulinemia, Immunodeficiency, and Myelokathexis syndrome (WHIM syndrome). The small molecule AMD3100 or Plerixafor, which was originally discovered as a potent antagonist of CXCR4 blocking HIV-1 entry in T-lymphocytes, is now market approved (trade name Mozobil) for hematopoietic stem cell mobilization. In combination with G-CSF, it is used in the treatment of non-Hodgkin lymphoma and multiple myeloma [10]. Below and in Table 4, an overview is provided of recent research investigating compounds that have reached clinical trials targeting the CXCL12-CXCR4/ACKR3 axis. On the other hand, studies are running in which CXCL12 could be locally delivered to promote tissue repair and wound healing.

**Table 4:** Overview of compounds inhibiting the CXCL12-CXCR4/ACKR3 interaction in recent in vivo experiments and in completed or ongoing clinical trials

Agent name (company)	Mechanism of action	Targeting diseases	In vivo model/Clinical trials	Results	Ref.
Plerixafor/AMD3100/Mozobil (Genzyme)	CXCR4 antagonist	Cervical cancer	Cervical cancer xenografts	Plerixafor with radiochemotherapy blunted therapy-induced increases in CXCL12 signaling & reduced tumor growth and intestinal toxicity	[266]
		Breast cancer	Triple negative breast cancer murine model	Liposomal-AMD3100 with immunotherapy reduced tumor size & prolonged survival	[267]
		Ovarian cancer	Ovarian cancer murine model	Plerixafor with immunotherapy inhibited tumor growth & prolonged survival by preventing multifaceted immunosuppression	[268]
		Multiple myeloma	NCT00903968 (Phase I/II: completed)	Plerixafor in combination with bortezomib is safe & the objective response rate is high	[326]
			NCT05087212 (Phase IV: recruiting)		
		Chronic lymphocytic leukemia	NCT00694590 (Phase I: completed)	Plerixafor is well-tolerated, supporting further studies	[272]
		Myelodysplastic syndromes	NCT01065129 (Phase I: completed)	Plerixafor with azacytidine chemotherapy is safe & demonstrated encouraging response rates	[273]
		Pediatric cancer	NCT01288573 (Phase I/II completed)	Plerixafor with standard G-CSF ± chemotherapy for HSC mobilization was well tolerated & efficacious	[274]
		Metastatic pancreatic cancer	NCT04177810 (Phase II: recruiting)		
		Glioblastoma	NCT03746080 (Phase II: recruiting)		
		WHIM syndrome	NCT00967785 (Phase I/II: recruiting)		
		COVID-19	NCT05411575 (Phase II: ongoing)		
			NCT04646603 (Phase II: recruiting)		
		Fanconi anemia	NCT02678533 (Phase I/II: completed)	HSC mobilization with plerixafor is safe & more efficient in younger patients without bone marrow failure	[284]
			NCT02931071 (Phase II: completed)	HSC mobilization with plerixafor is safe & efficient in pediatric patients	[285]
		Sickle cell disease	NCT03226691 (Phase I: completed)	Safe & sufficient collection of HSCs in most but not all participants. HSC mobilization is impacted by age, bone marrow reserve & disease severity	[286]
			NCT02193191 (Phase I: recruiting)		
			NCT03664830 (Phase I: recruiting)		
			NCT04817345 (Phase II: recruiting)		
			NCT05445128 (Phase II: recruiting)		
		HSC transplantation with matched sibling donors	NCT01696461 (Phase II: completed)	Rapid and safe mobilization of sufficient numbers of HSCs from matched sibling donors. Engraftment was prompt & outcomes in recipients were encouraging	[327]
		Chronic granulomatous disease	NCT03547830 (Phase II: recruiting)		
		Type 1 diabetes	NCT03182426 (Phase I/II: ongoing)		
		Idiopathic CD4 lymphocytopenia	NCT02015013 (Phase II: recruiting)		
Balixafortide/ POL6326 (Polyphor)	CXCR4 antagonist	Breast cancer	NCT03786094 (Phase III: ongoing)		
PTX-9908/CTCE-9908 (TCM Biotech)	CXCR4 antagonist	Hepatocellular carcinoma	NCT03812874 (Phase I/II: recruiting)		
Motixafortide/ BL-8040 (BioLineRx)	CXCR4 antagonist	Healthy volunteers	NCT05293171 (Phase I: ongoing)		
		Pancreatic cancer	NCT02826486 (Phase II: ongoing)	BL-8040 with immuno- and chemotherapy increased anti-tumor immune responses	[275]
			NCT02907099 (Phase II: ongoing)		
			NCT04543071 (Phase II: recruiting)		
			NCT03193190 (Phase I/II: recruiting)		
		Gastric & esophageal cancer	NCT03281369 (Phase I/II: recruiting)		
		Acute myeloid leukemia	NCT01838395 (Phase II: completed)	BL-8040 with chemotherapy increased survival & supported continued clinical development	[276]
		Multiple myeloma	NCT03246529 (Phase III: ongoing)		
		Advanced hematological cancer	NCT02639559 (Phase II: ongoing)		
X4P-001/mavorixafor/ AMD11070/AMD070 (X4 Pharmaceuticals)	CXCR4 antagonist	WHIM syndrome	NCT03005327 (Phase II: ongoing)	Mobilization of neutrophils and lymphocytes & reduced infections	[282]
			NCT03995108 (Phase III: ongoing)		
		Chronic neutropenia	NCT04154488 (Phase I: recruiting)		
		Renal cell carcinoma	NCT02923531 (Phase I/II: completed)	Manageable safety profile, supporting further studies	[328]
			NCT02667886 (Phase I/II: ongoing)		
		Waldenstrom’s macroglobulinemia	NCT04274738 (Phase I: ongoing)		
		Breast cancer	NCT05103917 (Phase I/II: recruiting)		
Spiegelmer NOX-A12/ Olaptesed (TME Pharma)	RNA-oligonucleotide binding CXCL12	Pancreatic & colorectal cancer	NCT03168139 (Phase I/II: completed)	NOX-A12 with immunotherapy was safe, supporting further clinical studies	[269]
		Chronic lymphocytic leukemia	NCT01486797 (Phase II: completed)	NOX-A12 with chemo- and immunotherapy was safe, supporting further clinical studies	[270]
		Multiple myeloma	NCT01521533 (Phase II: completed)	NOX-A12 with chemotherapy was safe, supporting further clinical studies	[271]
		Glioblastoma	NCT04121455 (Phase I/II: recruiting)		
ACT-1004–1239 (Idorsia)	ACKR3 antagonist	Healthy volunteers	NCT03869320 (Phase I: completed)	ACT-1004–1239 was safe & well tolerated supporting further clinical development	[279]
			NCT04286750 (Phase I: completed)	Favorable safety/tolerability and pharmacokinetic profiles, supporting further clinical development	[280]
Ulocuplumab/ BMS-936564/ MDX-1338 (Bristol-Myers Squibb)	Anti-CXCR4 antibodies	Waldenstrom’s macroglobulinemia	NCT03225716 (Phase I/II: ongoing)	Support for further clinical development	[277]
		Multiple myeloma	NCT01359657 (Phase I: completed)	Ulocuplumab is safe & leads to a high response rate if combined with lenalidomide and dexamethasone	[278]

This table provides an overview of compounds evaluated in recent (over the last 4 years) pre-clinical research, clinical trials with published results, and trials that are currently ongoing

*BRCA1* breast cancer-associated gene 1, *HSC* hematopoietic stem cell, *WHIM* Warts, Hypogammaglobulinemia, Immunodeficiency, and Myelokathexis syndrome

#### Targeting CXCL12-CXCR4/ACKR3 in cancer

Compounds aiming to block the angiogenic and chemotactic effect of CXCL12 to prevent tumor growth and metastasis are evaluated in pre-clinical and clinical research. Often, combinations of chemo-, radio-, or immunotherapy with a CXCR4 inhibitor (small molecules/monoclonal antibodies) or the RNA oligonucleotide spiegelmer NOX-A12 (which binds CXCL12 and thereby inhibits CXCL12-GAG interaction) are being evaluated. Indeed, as mentioned earlier, CXCL12 can induce resistance to chemo- or radiotherapy or create an immunosuppressive micro-environment. Application of a CXCL12/CXCR4 inhibitor (a) remodels and promotes release of adhesive tumor cells from the protective microenvironment making them more susceptible to conventional therapy and (b) improves the tumor infiltration and activation of cytotoxic T-lymphocytes in immune checkpoint blockade therapy [10]. Recent proof-of-concept studies were performed for breast, cervical, and ovarian cancer showing anti-tumor effects of CXCR4 antagonists (in combination with conventional therapy) in vivo (Table 4) [243, 266–268]. A number of recent clinical trials also showed promising results. Further clinical development is supported for the use of NOX-A12 together with conventional (immuno)therapy in colorectal and pancreatic cancer, chronic lymphocytic leukemia, and multiple myeloma [269–271]. Apart from the use of plerixafor in the treatment of non-Hodgkin lymphoma and multiple myeloma, it is currently being evaluated in combination with immuno- or radiochemotherapy in patients with metastatic pancreatic cancer or glioblastoma in phase II clinical trials. For chronic lymphocytic leukemia, myelodysplastic syndromes, and pediatric cancer it was already found to be safe and efficacious, supporting further studies [272–274]. Moreover, a novel CXCR4 antagonist, BL-8040 or motixafortide, in combination with immuno- and chemotherapy, increased anti-tumor immune responses in pancreatic cancer patients [275]. Other phase I/II trials for pancreatic cancer and phase II/III studies for hematological malignancies are currently ongoing with this compound. A combination of BL-8040 with chemotherapy already proved to be efficient in treatment of acute myeloid leukemia [276]. Finally, other CXCR4 antagonists are being evaluated in phase I to phase III clinical trials for treatment of renal cell and hepatocellular carcinoma, Waldenstrom’s macroglobulinemia, and breast cancer (Table 4). Interestingly, trials with a CXCR4-targeted antibody developed by Bristol-Myers Squibb, Ulocuplumab, supported further clinical development for use in Waldenstrom’s macroglobulinemia and multiple myeloma (Table 4) [277, 278]. Finally, besides CXCL12 or CXCR4, targeting ACKR3 showed promising pre-clinical results but had no running clinical trials in 2018 [10]. Now, randomized, placebo-controlled first-in-human studies with the novel small-molecule ACKR3 antagonist ACT-1004-1239 indicated that this compound was safe and well-tolerated, supporting further clinical development [279, 280].

#### Targeting CXCR4 in other (inflammatory) diseases

WHIM syndrome is a rare primary immunodeficiency disease characterized by severe neutropenia associated with frequent bacterial infections. It is caused by autosomal-dominant inherited gain-of-function mutations in the *CXCR4* gene, leading to CXCR4 hyperreactivity and impaired CXCR4 desensitization and internalization [10]. Recently, it was shown that defects in cell migration in WHIM syndrome patients may be caused by defects in the nanoclustering of the CXCR4 receptor at the cell membrane after CXCL12 activation, which is dependent on adequate β-arrestin 1 activation to induce remodeling of the actin cytoskeleton [281]. Obviously, CXCR4 antagonists and/or G-CSF could be useful to increase the circulating neutrophil counts in these patients. The oral small molecule CXCR4 antagonist X4P-001 (mavorixafor) induced efficient leukocyte mobilization and reduced infections and warts [282] and is currently being evaluated in a phase III trial (Table 4). For plerixafor evaluation in WHIM patients, a phase III clinical trial is completed but no results are published yet.

For COVID-19, two phase II clinical trials are ongoing for evaluation of the safety and efficacy of plerixafor or plerixafor in combination with a low dose of tacrolimus in hospitalized patients with COVID-19 ARDS. Mobilizing and recruiting stem cells/immunoregulatory cells might promote tissue regeneration and modulation of inflammation in severe COVID-19 [283]. Moreover, the stem cell mobilizing function of plerixafor is currently being investigated in phase I and phase II clinical trials for (autologous hematopoietic stem cell transplantation in) patients with sickle cell disease, chronic granulomatous disease, type 1 diabetes, and idiopathic CD4 lymphocytopenia (Table 4). It was already successful for mobilization in Fanconi anemia patients [284, 285] and first results for sickle cell disease patients indicate it is safe and efficient, but in some patients insufficient amounts of HSCs can be collected [286].

#### Using CXCL12 for tissue repair

Instead of blocking the CXCL12-CXCR4 interaction, the beneficial effects of CXCL12 itself could be used for therapeutic purposes. Indeed, topical administration of transformed lactic acid bacteria that locally produce recombinant CXCL12 accelerated wound healing. These bacteria have the additional and essential advantage that in the acidic environment CXCL12 is protected from CD26 proteolytic inactivation [287]. Recently, this method was found to be safe and accelerated healing using different evaluation methods in minipigs [288]. Over the last years, different research groups started investigating the use of hydrogels or scaffolds loaded with CXCL12 for local delivery of this chemokine to promote injury repair, wound healing, bone repair, and for cancer treatment. More specifically, CXCL12 could have potential in stem-cell therapy for treatment of traumatic brain injury. Indeed, in a cryogenic injury model in rats, an injectable hydrogel, loaded with human amniotic mesenchymal stromal cells and CXCL12, promoted migration and differentiation of these stem cells to nerve cells as such repairing focal brain injury [289]. A similar system promoted wound healing by stimulation of mesenchymal stem cell migration and secretion of pro-healing and regenerative cytokines [290]. A CXCL12/TGF-β1-loaded silk fibroin-porous gelatin scaffold, which provides a sustained release of CXCL12 and TGF-β1, promoted cartilage injury repair by facilitating cell homing and chondrogenic differentiation in vitro and in vivo [291]. Similar scaffolds/particles were also reported to induce stem cell migration promoting bone repair [292, 293] and intervertebral disc regeneration [294]. Finally, a similar mechanism attracted and trapped circulating murine CXCR4-expressing melanoma tumor cells in vitro and in vivo after subcutaneous injection, thereby reducing lung metastasis [295].

## Concluding remarks

In this review, the pivotal roles of the chemokines CXCL8 and CXCL12 in health and disease were discussed, focusing on their structure, receptor and GAG interactions, activity regulation, and therapeutic perspectives. While CXCL8 exclusively plays a role in inflamed conditions and is often one of the most abundant chemokines at the onset of inflammation, CXCL12 exerts both homeostatic as well as inflammatory functions dependent on the (patho)physiological context. The structural interactions with receptors and GAGs and activation of specific downstream G protein- or β-arrestin-mediated signaling pathways by chemokine or receptor monomers and homo- or heterodimers is for both CXCL8 and CXCL12 far from completely elucidated. However, the continuously growing list of pathologies in which these chemokines are involved, highlights the significance of further research on function and activity regulation of this system. Although differences between mice and men for CXCL8 do not facilitate this research, increasing knowledge in this field and improved techniques open new windows for identification of novel and improved drug targets. However, further understanding of (a) the spatiotemporal control of chemokine activity, (b) the protective or detrimental role of CXCL8 and CXCL12 signaling in specific diseases and (c) the detection of CXCL8 and CXCL12 isoforms and proteoforms with specific activities, which cannot be discriminated by most standard immunoassays using antibodies, will be required to determine appropriate drug targets. As such, novel therapies targeting the chemokine system, in combination with classical (immuno) therapy, could have great potential in cancer and inflammatory diseases.
